# Carcinoma-associated fucosylated antigens are markers of the epithelial state and can contribute to cell adhesion through *CLEC17A* (Prolectin)

**DOI:** 10.18632/oncotarget.7476

**Published:** 2016-02-18

**Authors:** Adrien Breiman, María Dolores López Robles, Sophie de Carné Trécesson, Klara Echasserieau, Karine Bernardeau, Kurt Drickamer, Anne Imberty, Sophie Barillé-Nion, Frédéric Altare, Jacques Le Pendu

**Affiliations:** ^1^ Inserm U892, CNRS UMR6299, University of Nantes, 44007 Nantes, France; ^2^ Nantes University Hospital, 44007 Nantes, France; ^3^ Department of Life Sciences, Imperial College London, London SW7, UK; ^4^ CERMAV-UPR 5301, CNRS, Université Grenoble Alpes, 38041 Grenoble, France; ^5^ Recombinant Protein Core Facility of The University of Nantes, 44007 Nantes, France

**Keywords:** fucose, lectins, epithelial to mesenchymal transition, lymph node metastasis

## Abstract

Terminal fucosylated motifs of glycoproteins and glycolipid chains are often altered in cancer cells. We investigated the link between fucosylation changes and critical steps in cancer progression: epithelial-to-mesenchymal transition (EMT) and lymph node metastasis.

Using mammary cell lines, we demonstrate that during EMT, expression of some fucosylated antigens (e.g.: Lewis Y) is decreased as a result of repression of the fucosyltransferase genes *FUT1* and *FUT3*. Moreover, we identify the fucose-binding bacterial lectin BC2L-C-Nt as a specific probe for the epithelial state.

Prolectin (CLEC17A), a human lectin found on lymph node B cells, shares ligand specificities with BC2L-C-Nt. It binds preferentially to epithelial rather than to mesenchymal cells, and microfluidic experiments showed that prolectin behaves as a cell adhesion molecule for epithelial cells. Comparison of paired primary tumors/lymph node metastases revealed an increase of prolectin staining in metastasis and high *FUT1* and *FUT3* mRNA expression was associated with poor prognosis. Our data suggest that tumor cells invading the lymph nodes and expressing fucosylated motifs associated with the epithelial state could use prolectin as a colonization factor.

## INTRODUCTION

Alterations of glycosylation are a hallmark of cancer. In particular, N- or O-glycans of proteins as well as glycolipids produced by cancer cells often carry an increased number of fucosylated motifs of the Lewis blood groups family [1, 2]. Elevated levels of Lewis Y (Le^y^), Sialyl-Lewis A (SLe^a^) or Sialyl-Le^x^ (SLe^x^) have been found in numerous types of carcinomas including colon or breast. Expression of such motifs has been shown to be associated with disturbance of the cell adhesion properties, increase in the migration capacity and invasion of ectopic tissues [3–5]. Although sialylated Lewis antigens have clearly been demonstrated to favor cancer cells extravasation through their interaction with selectins expressed on leukocytes or endothelial cells, functional consequences of neutral fucosylated antigens overexpression have remained elusive. Before entering the circulation, carcinoma cells go through a reversible cellular process called epithelial-to-mesenchymal transition (EMT). EMT occurs during normal development and is also a hallmark of cancers. It consists in the loss of cell-cell contact and acquisition of mobility and invasiveness properties and has been involved in metastasis. EMT is characterized by a decrease in the expression of adhesion-related proteins (typically E-Cadherin) and increased expression level of various factors such as the cytoskeletal protein vimentin. The genetic reprogramming accompanying EMT is regulated by various transcription factors, belonging to 3 main families (Snail, Zeb and Twist) as well as by miRNAs, especially from the miR200 family, and can be triggered by cytokines such as TGF-β. Generation of cancer stem cells (CSC) has also been associated with EMT (reviewed in [6]). It is generally assumed that in order to colonize foreign tissue and establish metastasis, cells have to go through the reverse mechanism, mesenchymal-to-epithelial transition (MET), however this process has not received as much attention as EMT.

As an alternative to the use of antibodies for the detection of abnormal glycosylations in cancer patients, proteins with carbohydrate-binding properties called lectins have been investigated. Although they were first identified in plants, lectins are present in all kinds of organisms from bacteria to humans. They are grouped in families according to their structural characteristics, chemical properties and ligand specificities [7]. Several lectins have been found to have relevance for cancer prognosis, including a lectin of the edible snail (*Helix pomatia*) and a lectin from the plant *Maclura pomifera*. Binding of those two lectins correlates with bad prognosis for breast and colon carcinomas [4, 8]. We recently identified the fungal lectin from *Psathyrella velutina* as a marker of truncated N-glycans present on lung cancer cells [9]. However use of lectins in the clinic has been limited until now, partly because of the difficulties associated with reliable production of lectins from plants or animals.

While lectins from plants, mushrooms or invertebrates have low affinity for human glycans, lectins from opportunistic bacteria, that have evolved to use human glycans as targets, could present stronger affinity and be used as specific markers. In the recent years, the α-galactose (Gal) specific LecA from *Pseudomonas aeruginosa*, the α-mannose (Man) specific Bc2L-A from *Burkholderia cenocepacia* and the α-fucose (Fuc) specific RSL from *Ralstonia solanacearum* were produced in recombinant form and described [10–12]. Of special interest, the BC2L-C-Nt lectin from *B. cenocepacia* has been successfully produced in a recombinant manner. It folds in a trimeric TNF-α-like structure and binds to α2-fucosylated blood group antigens such as H type 1/3 or Le^y^ [13, 14].

In this study we investigated the evolution and possible roles of fucosylated antigens expression during cancer progression. We used a panel of antibodies and lectins targeting Lewis antigens and found an association between expression of these antigens and the epithelial state, expression being lost in the mesenchymal state. We show that BC2L-C-Nt is a good tool to monitor these changes.

Since some mammalian lectins, belonging to the calcium-dependant family (C-type lectins), are able to bind Lewis fucosylated antigens, we considered the possibility that endogenous lectins could play a role in tissue colonization *via* interaction with tumor cells after they have engaged in MET. Indeed, C-type lectins play a role in processes such as cell-adhesion, leucocyte extravasation and pathogen recognition [2, 15]. Our observation of a link between the epithelial state and expression of fucosylated glycans revealed using the BC2L-C-Nt bacterial lectin prompted us to look for potential endogenous lectins with similar glycan specificity. One intriguing member of this family is prolectin (encoded by the *CLEC17A* gene), which seems to be expressed mainly in dividing B cells found in the germinal centers of secondary lymphoid organs. Prolectin is a type II membrane protein with an extracellular carbohydrate-recognition domain (CRD) closely resembling the CRD of the well-characterized dendritic cell lectin DC-SIGN. However, the exact function of prolectin remains unknown [16]. Here we show that Prolectin can serve as a cell adhesion molecule for fucosylated epithelial cancer cells. We suggest a model presenting a possible role of prolectin in implantation of metastases in lymph nodes.

## RESULTS

### Epithelial cells express more fucosylated antigens than mesenchymal cells

**Figure 1:**
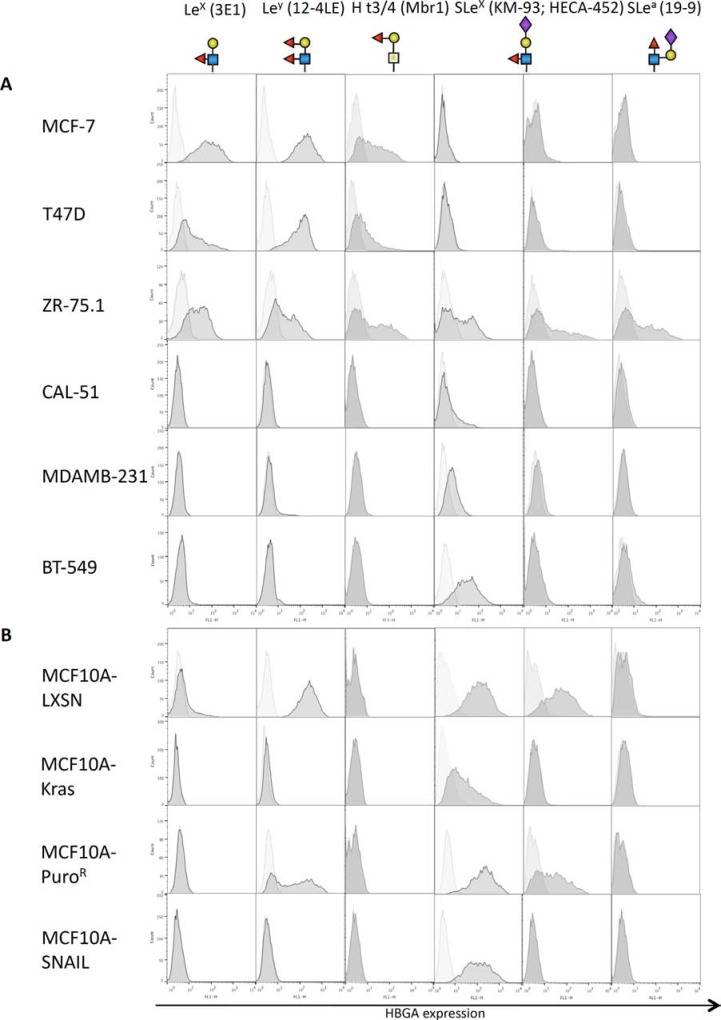
Expression of fucosylated antigens by mammary cell lines Breast cell lines from tumor origin (**A**) or derived from the immortalized MCF10A cell line (**B**) were subjected to flow cytometry using various antibodies directed against fucosylated histo-blood groups antigens, followed by an anti-mouse-FITC secondary antibody. The horizontal axis represents mean fluorescence intensity (MFI) while cell count is indicated on the vertical axis. Structures of the glycan epitopes recognized by each antibody are shown below the antibody description.

EMT is characterized by a profound reprogramming of cellular gene expression. We thus sought to identify differences in histo-blood group antigens (HBGAs) displayed on the membranes of epithelial and mesenchymal cells (See Figure S1 for a diagram of HBGA synthesis pathways). We worked on breast cancer cell lines for which the EMT status has been well described. In addition, we used two EMT models based on the immortalized epithelial breast cell line MCF10A, from which mesenchymal counterparts had been derived by transfection with EMT-inducing factors, respectively the constitutively active oncogene Kras(v12) and the transcription factor SNAIL (*SNAI1* gene). The control cell lines transfected with empty vectors and selected in parallel of MCF10A-KRAS(v12) and MCF10A-SNAIL are referred to thereafter as MCF10A-LXSN and MCF10A-Puro^R^ respectively. We looked at the expression of several cancer-associated fucosylated antigens using flow cytometry and appropriate mouse mAbs (Figure 1A and 1B). All epithelial breast cell lines were found to express Le^y^ as well as Le^x^ and H type 3 antigens, except the non-cancerous cell lines MCF10A-LXSN/MCF10A-Puro^R^ that expressed only Le^y^. The slight difference in Le^y^ expression profile between the two MCF10A control cell lines is probably due to clonal selection. Nonetheless, none of the neutral fucosylated antigens were detected on mesenchymal cell lines, including MCF10A-Kras(v12) and MCF10A-SNAIL. Some epithelial (MCF10A-LXSN, MCF10A-Puro^R^, ZR-75.1) as well as mesenchymal cell lines (BT-549, MDA-MB-231, MCF10A-KRAS(v12), MCF10A-SNAIL) were positive for SLe^x^ expression detected by the KM-93 antibody. However the HECA-452 antibody that is more fucose dependent than the KM-93 [17] only stained epithelial cell lines (ZR75.1, MCF10A-LXSN and MCF10A-Puro^R^). SLe^a^ was poorly expressed if at all on the breast cell lines tested except for ZR75.1.

### BC2L-C-Nt preferentially binds to epithelial breast cell lines

We then tested fucose-specific bacterial lectins: BC2L-C-Nt from *B. cenocepacia* and the broadly fucose-specific RSL from *R. solanacearum.* As a comparison, we also tested the well-characterized α2-fucose-specific plant lectin *Ulex europeaus agglutinin-I* (UEA-I). Lectins used are listed in Table S1.

**Figure 2:**
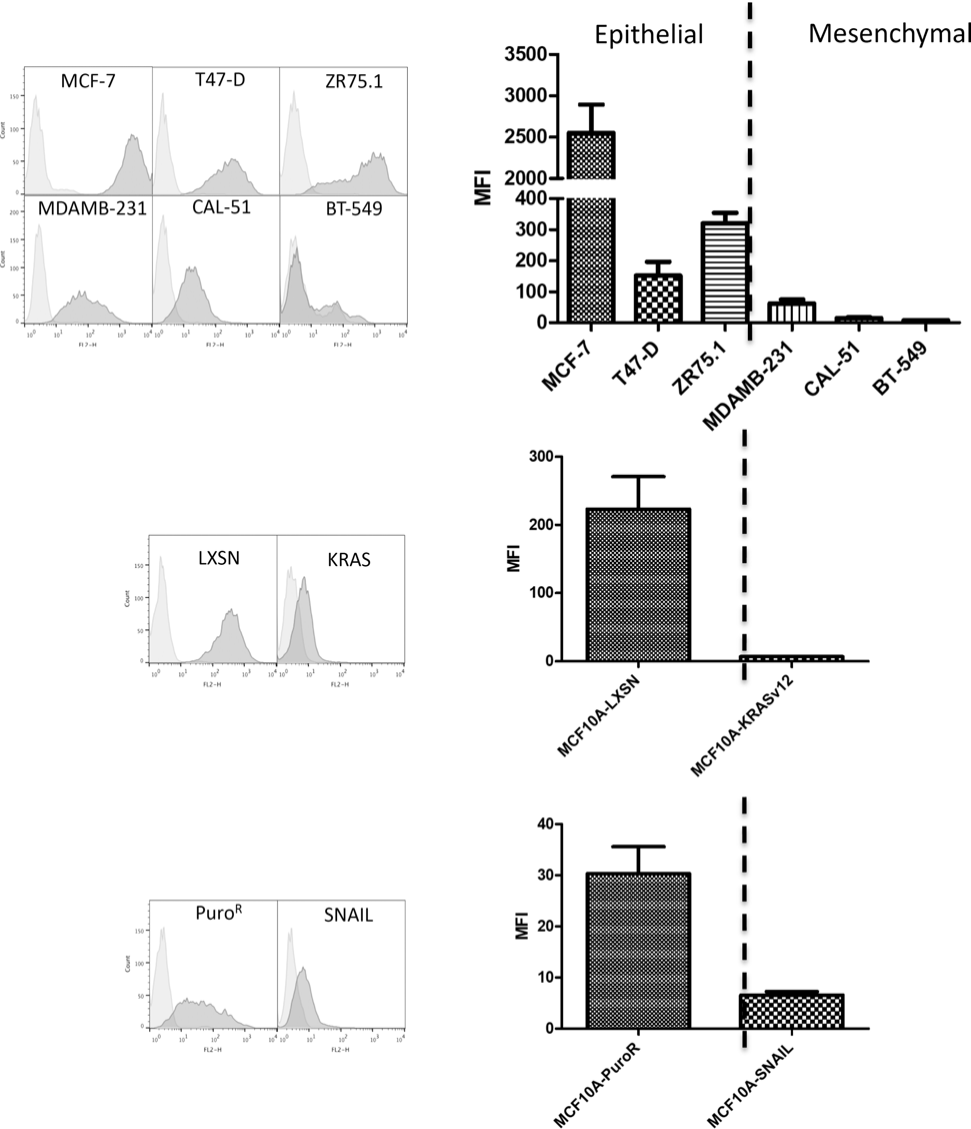
The fucose binding lectin BC2L-C-Nt stains epithelial cells rather than mesenchymal cells Breast cell lines were stained with biotinylated lectins BC2L-C-Nt followed by PE-conjugated streptavidin and analyzed by flow cytometry. Representative flow cytometry histograms as well as a histogram of the mean and SEM of Mean fluorescence intensities (MFI) for three independent experiments are presented.

We found that the BC2L-C-Nt staining was clearly higher on cell lines characterized as epithelial as compared to those characterized as mesenchymal (Figure 2). Interestingly, other fucose-specific lectins such as UEA-I and RSL did not distinguish between the two phenotypes as clearly as BC2L-C-Nt (Figure S2A). In order to determine if lectins with other types of specificity could present a similar or complementary binding profiles compared to BC2L-C-Nt, we tested BC2L-A, and LecA, specific for mannose and galactose respectively and neither of these lectins showed a clear distinction between epithelial and mesenchymal cells (Figure S2B).

### Epithelial cells express higher levels of FUT1 and FUT3 than mesenchymal cells

**Figure 3:**
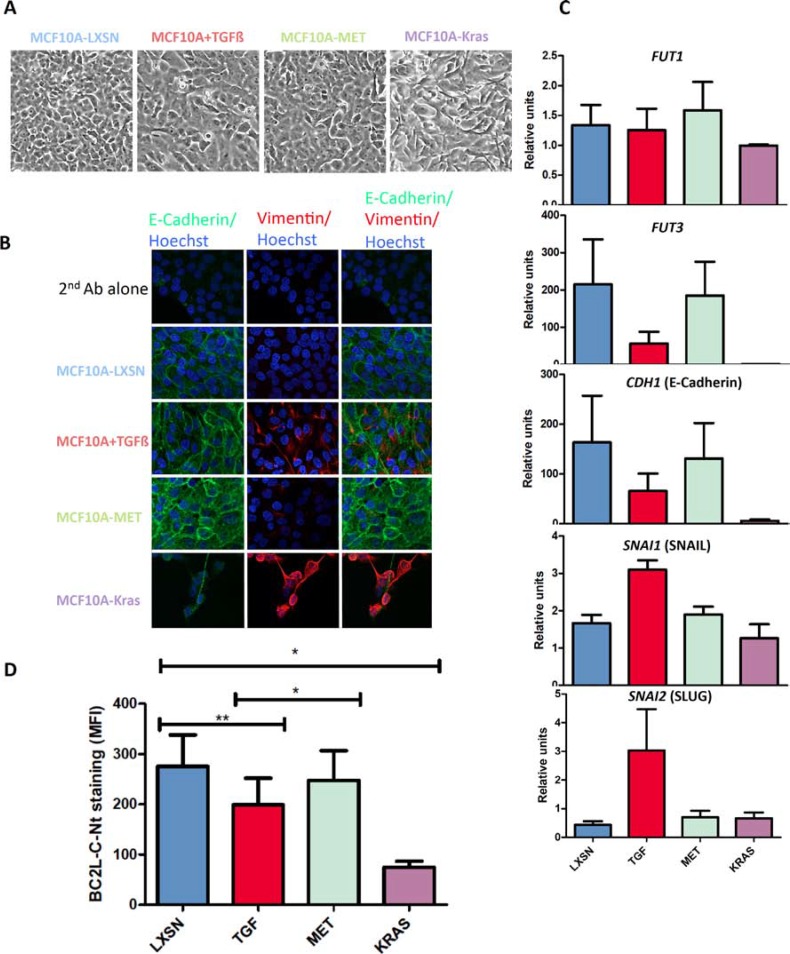
BC2L-C-Nt staining decreases following EMT induction in parallel with fucosyltransferase expression MCF10A-LXSN cells were passaged in the presence of 10 ng/ml TGF-Δ1 e-Bioscience to induce EMT (MCF10A+TGF-Δ). The cells were then grown a further 4 days in the absence of TGF-Δ to induce a MET (MCF10A-MET). (**A**) Morphological appearance of the cells. (**B**) MCF10A-LXSN epithelial cells were treated with either 0,5 μM A83-01 (MCF10A-LXSN) or 5 ng/ml TGF-Δ1 R & D (MCF10A+TGF) during four days. TGF-Δ was then withdrawn and cells were grown four more days in presence of 0,5 μM A83-01 (MCF10A-MET). Cells were analyzed by immunofluorescence using anti-E-cadherin and anti-Vimentin antibodies followed respectively by a Dylight 488-conjugated and a Rhodamine-conjugated secondary antibody. Nuclei were stained with Hoechst. Pictures were taken with a Nikon confocal microscope at a x60 magnification (**C**) Total RNAs were extracted from the different cell lines and cDNAs were synthesized using oligo-dT and subjected to qPCR analysis with *FUT1, FUT3* and *GAPDH* specific primers and probes. *FUT1* and *FUT3* expression levels are presented relative to *GAPDH* expression. The PCR on *CDH1, SNAI1 and SNAI2* was performed using specific primers and SYBrGreen and results are presented relatively to Δ*-actin* expression. Data shown are the mean and SEM of three independent experiments. (**D**) Binding of BC2L-C-Nt to cells analyzed by flow cytometry. The results are the means and SEM of the MFI of BC2L-C-Nt staining for four independent experiments including the three used in Figure 3C. Statistical significance has been assessed with 2-tailed paired *t*-tests between the indicated data sets.

In order to confirm the epithelial preference of BC2L-C-Nt, we treated MCF10A cells with TGF-Δ, which was able to induce a partial EMT as shown by: (1) morphological change (Figure 3A), (2) vimentin induction as shown by immunofluorescence (Figure 3B) and flow cytometry (Figure S3) and (3C) a reduction of E-cadherin mRNA expression and an increase of SNAIL and SLUG mRNA expression (See Figure 3C). We performed FACS analysis with anti-Le^y^ and anti-SLe^x^ on TGF-Δ treated cell. Although we could not observe a decrease in the 12-4LE staining, KM-93 and HECA-45 labeling were reduced by respectively 40 and 70% (Figure S3). We also found a small but significant reduction in BC2L-C-Nt staining in cells treated with TGF-Δ (Figure 3D). Reversion to the epithelial phenotype (MET) could be partially achieved by withdrawing TGF-β (as shown by a reduction of vimentin expression as well as by increase of E-Cadherin and decrease of SNAIL and SLUG mRNAs levels, See Figure 3B and 3C) and this was accompanied by an increase in BC2L-C-Nt staining (Figure 3D). We then investigated whether the difference in fucosylated antigen expression between epithelial and mesenchymal cell lines reflected a difference in fucosyltransferase gene expression. We first analyzed available transcriptomic data from a panel of 51 breast cancer cell lines [18]. Breast cell lines have been classified in categories called luminal, corresponding to the luminal A/B tumour histologic type and associated with a pronounced epithelial phenotype, Basal A, corresponding to the Basal tumor type and Basal B, characterized by a high expression of mesenchymal specific genes and CSC markers [19, 20]. In humans, 8 fucosyltransferases genes can contribute to the synthesis of fucosylated antigens: *FUT1-7* and *FUT9*. *FUT1* and *FUT2* encode α 1–2-fucosyltransferases, whereas the others encode α 1–3/4-fucosyltransferases [21]. We observed that *FUT5, 6, 7* and *9* were weakly or very weakly expressed in the breast cell lines. *FUT1* and *FUT3* were significantly more expressed in luminal and Basal A cell lines than in the Basal B cell lines, whereas no significant difference was observed for *FUT2* and *FUT4* (Figure S4). We therefore used the MCF10A model to check if there is a relationship between *FUT1/FUT3* expression and EMT. We performed qRT-PCR using *FUT1*- and *FUT3*-specific probes on untreated or TGF-Δ-treated MCF10A-LXSN cells, MCF10A-Kras(v12) cells and MCF10A-Puro^R^/MCF10A-SNAIL cells. We observed that *FUT3* expression was markedly reduced when EMT was induced by TGF-Δ treatment. In addition, *FUT3* expression was at least partially restored upon TGF-Δ withdrawal. Moreover *FUT3* was absent in cells overexpressing Kras(v12) or SNAIL. The *FUT3* gene expression pattern was strikingly similar to that of the *CDH1* gene encoding E-cadherin, but in contrast with that of *SNAI1* (SNAIL) and *SNAI2* (SLUG) (Figure 3C and Figure S5). The association between *FUT1* and EMT was less pronounced (Figure 3C), although its expression was suppressed by SNAIL overexpression (Figure S5). The above results prompted us to look at the promoter sequences of the *FUT1* and *FUT3* genes to see if there were binding motifs for the EMT inducing transcription factors. We found several E2-boxes (CAGGTG or CACCTG), which are classical SNAIL-binding motifs, similar to those that can be found in the E-cadherin promoter. Moreover, bipartite elements (C)AGGTG/CACCT(G) are binding motifs for ZEB1/2, which are also EMT-inducing transcription factors [22, 23] (Figure S6A). Analysis of the transcriptome of the 51 breast cancer cell lines showed an inverse relationship between expression of *FUT1/3* and of *SNAI2* and *ZEB1/2* (Figure S6B).

The above results from surface fucosylation staining and analysis of fucosyltransferase expression suggest that there is a relationship between surface expression of neutral α1, 2 and α1, 3/4 fucosylated glycans, and EMT/MET.

### The human C-type lectin prolectin has similar glycan specificity as BC2L-C-Nt and efficiently binds human cell lines and tumor tissues

The association between the epithelial state and fucosylated glycans that we demonstrated above could result in interaction with some endogenous lectin(s) in the metastatic microenvironment that could favor tumor cells implantation. We identified in databases the fucose-specific lectin CLEC17A (prolectin) as a good candidate because it recognizes non-sialylated Lewis-type ligands and has the interesting property of being expressed mainly in secondary lymphoid organs, a very common site for tumor metastasis.

**Figure 4:**
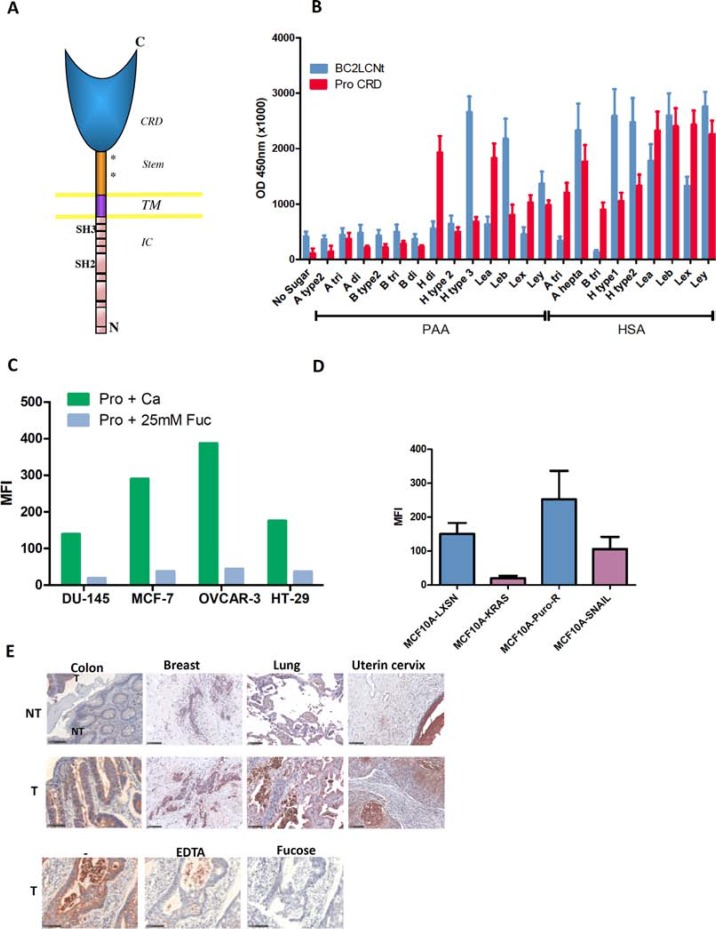
Prolectin is a human C-type lectin that recognizes similar fucosylated ligands as BC2L-C-Nt and efficiently binds to tumor cell lines and tumor tissues (**A**) Diagram of Prolectin structure (CRD: carbohydrate recognition domain; TM: transmembrane domain; IC: intra-cytoplasmic domain). (**B**) Glycan binding assay of prolectin and BC2L-C-Nt. Plates coated with a series of fucosylated HBGAs conjugated to HSA or PAA were probed with either biotinylated-BC2L-C-Nt followed by Avidin-HRP or tetramers of biotin-prolectin CRD bound to streptavidin-HRP. The results shown are the mean and SEM of three independent experiments. (**C**) Flow cytometry staining of tumor cell lines from various origins. Tetramers of biotinylated-prolectin CRD in complex with streptavidin-PE, pre-incubated in the absence of sugar or with L-fucose, were bound to the indicated cell lines, which were then analyzed by flow cytometry. The bar histogram shows the mean fluorescence intensities. Results are representative of at least two experiments. (**D**) Prolectin preferentially stains epithelial cell lines. The epithelial/mesenchymal cell lines pairs MCF10A-LXSN/MCF10A-Kras(v12) and MCF10A-Puro^R^/MCF10A-SNAIL were stained with prolectin as described above. Means and SEM of three independent experiments are shown (**E**) Prolectin binding to tumor tissues. Tetramers of biotinylated-prolectin CRD conjugated with streptavidin-HRP were incubated on fixed tissue sections from different tumor (T) or adjacent normal (NT) tissues. To control for the involvement of specific glycan binding in the prolectin staining, colon tumor sections were stained as above after prolectin tetramers were incubated with Ca^2+^, EDTA or Ca^2+^+ L-fucose. Colon tissues had been fixed in ethanol whereas breast, lung and uterine tissues were from formalin-fixed TMAs. Magnifications of 20× are shown (the black bars represent 100 μm).

Prolectin is a type II transmembrane C-type lectin with an extracellular CRD and a cytoplasmic tail containing SH2 and SH3 signaling domains (Figure 4A). It has been shown to bind both poly-mannosylated ligands and neutral fucosylated ligands on the glycan microarray from the Consortium for Functional Glycomics [16].

The prolectin CRD was produced in bacterial cells, purified by gel filtration, biotinylated *in vitro* and used in a tetrameric complex with streptavidin as described in Materials and Methods. We then tested prolectin binding on a range of fucosylated ligands of the HBGA series. As lectin binding to carbohydrate is quite dependent on the underlying molecule, we used two different kinds of carriers: polyacrylamide beads (PAA) or human serum albumine (HSA).

When we compared prolectin and BC2L-C-Nt binding on a range of neutral HBGAs, the two lectins showed quite similar but distinct binding profiles. In particular, Le^y^ was one of the most efficiently recognized ligands for both of these lectins (Figure 4B; detailed formulas of the oligosaccharides used are given in Table S3). We went on to test prolectin binding to a panel of established tumor cell lines from various histological origins: breast, colon, ovary, prostate, which were all positive for Le^x^ and Le^y^ (Table S4). Prolectin was found to bind to all the tumor cell lines. As expected, this interaction is mediated by the sugar-binding site, as it was abolished by pre-incubation of the lectin with an excess of fucose monosaccharide (Figure 4C).

We also tested prolectin binding on the MCF10A cell lines and found that the fluorescence level on MCF10A-LXSN and MCF10A-Puro^R^ cells was markedly higher than on the Kras(v12)- and SNAIL-transfected counterparts, showing that, like BC2L-C-Nt (but to a lesser extent), prolectin displays a preference for epithelial cells (Figure 4D).

We tested prolectin binding to human tumor tissue sections from breast (*N* = 40), colon (*N* = 39), lung (*N* = 39) and uterine cervix (*N* = 50). Prolectin stained 80–95% of the tumors (≥ 5% of positive cancer cells). The percentage of cancer cells stained was quite variable (between 5 and 100%) for breast and uterine tumors. Most lung and colon tumors presented ≥ 80% positive cells. We also noticed in some tumor samples a high number of infiltrating leucocytes that were strongly stained, although lymphoid tissues were mostly negative (Figure S7). Paired adjacent healthy tissue was generally much less stained that the corresponding tumor and the labeled structures were mostly the outer epithelial layers, preferentially on the apical side (Figure 4E). Again, this binding of prolectin was glycan dependent, as it was inhibited in the presence of EDTA, which chelates the Ca^2+^ of the CRD as well as in the presence of fucose (Figure 4E).

### Prolectin can behave as an adhesion molecule for cells expressing fucosylated ligands

Since prolectin is a membrane C-type lectin, an interesting hypothesis was that it could mediate interactions with cells expressing the appropriate ligands, as selectins do with SLe^x^ expressing cells. We decided to investigate this possibility using the BioFlux microfluidic system. This system makes it possible to generate a tightly controlled shear flow pressure inside microfluidic channels, which can be coated with cells or proteins. As prolectin is expressed on B cells in the lymph nodes, the interaction with tumor cells is not expected to happen in the blood stream (in contrast to the interaction with selectins), however we thought that performing our experiments under a low shear flow (0,05–0.1 Dyn/cm^2^) would correspond to the situation of motile cells reaching lymphoid organ cells and would additionally give higher stringency for the study of cell-prolectin interaction than using static conditions.

**Figure 5:**
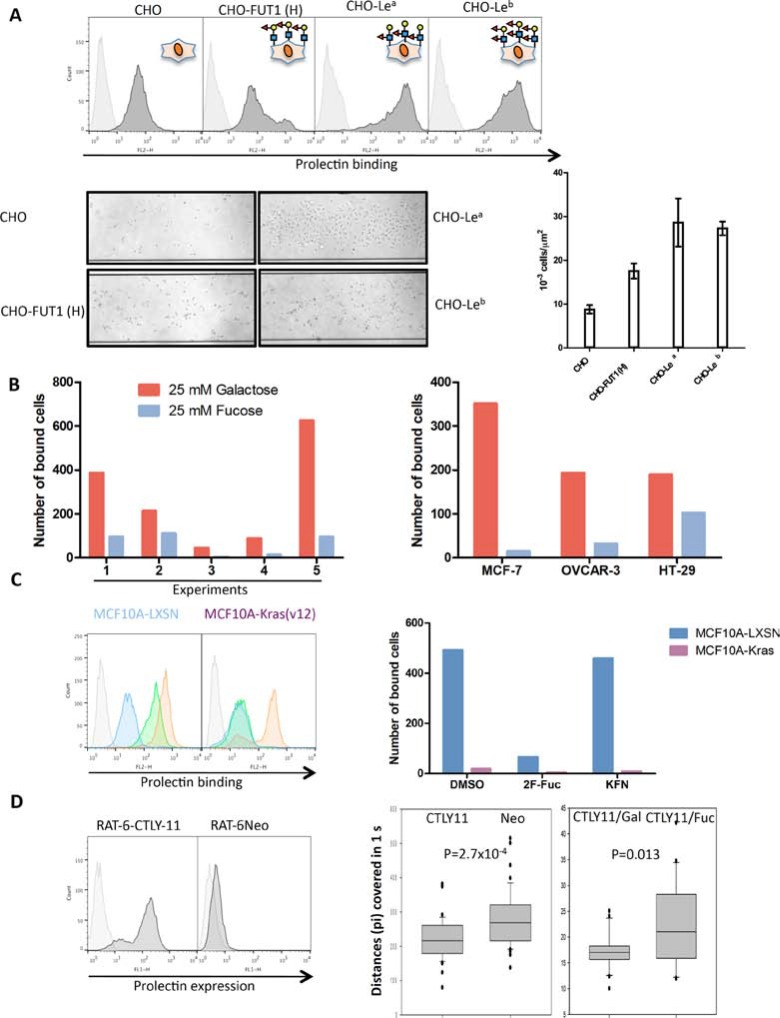
Prolectin acts as a cell adhesion molecule (**A**) Expression of fucosylated markers on normal CHO cells or CHO cells engineered to express fucosylated antigens tested by flow cytometry. Cells were introduced in the channels of a BioFlux microfluidic system coated with prolectin/streptavidin tetramers under a pressure of 0.1 Dyn/cm^2^. Unbound cells were washed away and pictures of the attached cells were taken at 10x magnification with a Leica DMI 6000B microscope. Bound cells were counted on three different segments along the channels and the mean cell densities and SEM are represented on the histogram. (**B**) DU-145 cells were introduced in the channels under a pressure of 0.1 Dyn/cm^2^ and in presence or 25 mM D-galactose or L-fucose. Unbound cells were washed away and pictures of the attached cells were taken. Bound cells were counted in the whole channels. The results of five independent experiments are shown. Due to the high variation between experiments, the results are shown separately. Similar experiments were performed with the other tumor cell lines MCF-7, OVCAR-3 and HT-29. (**C**) MCF10A-LXSN and MCF10A-Kras(v12) cells were treated with dimethyl sulfoxide (DMSO, green), 5 μM kifunensin (orange), or 400 μM 2F-fucose (blue) during four days and stained with prolectin by flow cytometry as described above. Control cells and cells treated with the inhibitors were injected into prolectin-coated BioFlux channels. Unbound cells were washed away and pictures of the attached cells were taken. Bound cells were counted in the whole channels. (**D**) Prolectin expression was tested on Rat6-Neo and Rat6-CTLY11 fibroblasts by flow cytometry. Percentages of “positive” cells are indicated. Rat6-Neo and Rat6-CTLY11 or Rat6-CTLY11 in the presence of 25 mM galactose or fucose were injected in BioFlux channels covered with a layer of DU-145 cells under a pressure of 0.05 Dyn/cm^2^. Films of 1 minute (600 pictures) were acquired on a Leica DMI 6000B microscope using the Metamorph software. Chronophotographic images, corresponding to 10 pictures segments of the films, were constructed using the FiJi software and distances covered by individual cells were measured. Student tests were performed to assess statistical significance.

We coated the microchannels with prolectin CRD/streptavidin tetramers and injected Chinese hamster ovary (CHO) cells transfected with one or several glycosyltransferases so as to make them express various fucosylated ligands (H type 2, Le^a^, Le^b^; Figure S8). A few normal CHO cells bound to the prolectin-coated surface, however twice as many of the CHO-H (FUT1) and 3-times more of the CHO-Le^a^ and CHO-Le^b^ were bound, consistent with the prolectin staining of the cells as analyzed by flow cytometry (Figure 5A). Interestingly, after less than 30 mn of binding, the CHO-Le^a^ cells already displayed an elongated shape. We thus compared CHO and CHO-Le^a^ binding on Lab-tek chamber slides coated with either prolectin or poly-lysine. After 2 h, bound CHO cells had a round shape regardless of which coating was used. CHO-Le^a^ cells were mostly round or ovoid on poly-lysine, but most were elongated when bound on the prolectin coating (Figure S9).

We next tested the binding of human cancer cell lines on the prolectin coating under flow conditions. To this end, we injected the cells in a buffer containing 25 mM of either fucose or galactose. We performed 5 independent experiments with the prostate cancer cell line DU-145, which has good characteristics for this kind of experiments, as the trypsinized cells are round and mostly well isolated. Despite important variations in the absolute number of bound cells, there were always more bound cells in presence of galactose, which should not affect prolectin, compared to the presence of fucose, which blocks prolectin. This pattern was also observed with the other cancer cell lines MCF-7, OVCAR-3 and HT-29 (Figure 5B). The affinity of prolectin galactose is weak but not inexistent (see [16]), which means that the efficiency of adhesion of those two cell lines to the prolectin coating might have been somewhat underestimated in our experimental design. We also found that bound cells were those expressing the highest level of prolectin ligands (Figure S10).

Binding of MCF10A-LXSN and MCF10A-Kras(v12) cells to a prolectin coating was also compared using the BioFlux system. Flow cytometry analysis was performed in parallel. As already mentioned, prolectin stained MCF10A-LXSN cells more strongly than MCF10A-Kras(v12) cells. An even more important difference was found when looking at adhesion of cells to the prolectin layer under the shear flow conditions, since only the epithelial MCF10A-LXSN cells, but not their Kras(v12) mesenchymal counterparts, were able to adhere (Figure 5C). Prolectin recognizes fucosylated ligands, but also high mannose glycans [18]. Thus, in order to get better insight into which type of sugar ligands were involved, we treated cells with glycosylation inhibitors. First, we used 2-fluoro-fucose (2F-Fuc), a competitive inhibitor of all the cellular fucosyltransferases. The effect of 2F-Fuc treatment on the expression of the fucosylated markers was controlled using BC2L-C-Nter. The labeling of treated cells was nearly completely abolished (Figure S11). 2F-Fuc treatment markedly decreased prolectin binding to MCF10A-LXSN cells while it did not have any effect on the lower binding to MCF10A-Kras(v12) cells, strongly suggesting that the difference in staining by prolectin between the two cell lines is essentially due to the presence of fucose residues on MCF10A-LXSN cells. Moreover, 2F-Fuc treatment suppressed adhesion of MCF10A-LXSN cells to the prolectin coating, showing that fucose residues are primarily responsible for the binding (Figure 5C). To further delineate the respective role of fucose versus mannose recognition by prolectin, we also treated the cells with kifunensine (KFN), an α-mannosidase I inhibitor that blocks maturation of N-glycan chains maturation and consequently increases expression of high mannose chains at the cell surface. The increase of mannose presentation by KFN was controlled by staining with the mannose-specific lectin BC2L-A (Figure S11). As expected, KFN treatment increased prolectin binding on both cell types. However, KFN did not change the efficiency of adhesion of these cells to the prolectin layer (Figure 5C). This result confirmed that fucosylated ligands play a major role in prolectin binding to epithelial cells, while mannose residues constitute the remaining ligands on mesenchymal cells. However, mannose recognition did not appear to significantly contribute to adhesion of prolectin, which was mainly fucose-dependent.

Since a coating of the prolectin CRD was able to mediate adhesion of cells under flow, we sought to determine whether adhesion would also happen in a cell-cell interaction context with the native protein. For these experiments, we used RAT6 rat fibroblasts transduced with a retrovirus containing the whole *CTLY11* (*CLEC17A*) prolectin cDNA. About half of the RAT6-CTLY11 cells were positive for surface prolectin expression whereas no prolectin was detected on the cells transduced with the empty vector (RAT6-Neo) (Figure 5D).

A monolayer of DU-145 cells was grown in the BioFlux channel and RAT6-Neo or RAT6-CTLY11 cells were injected. We compared the speed of movement of RAT6-Neo and RAT6-CTLY11 cells by capturing short videos and measuring the distance covered by the fibroblasts on image sequences corresponding to 1 second. We found that prolectin-expressing fibroblasts passed through the channel more slowly than the RAT6-Neo cells. We then injected RAT6-CTLY11 suspended in a buffer containing either galactose or fucose. Cells were found to move significantly more slowly in the presence of galactose than in the presence of fucose (Figure 5D). Similar results were obtained on a monolayer of MCF-7 cells (Figure S12). Although involvement of other fucose-specific lectins cannot be formally excluded, these results suggest that the presence of prolectin at the cell surface can induce rolling onto a layer of tumor cells.

### Higher levels of prolectin ligands are associated with lymph node metastasis and elevated FUT1/3 expression correlates with a worse prognosis

**Figure 6:**
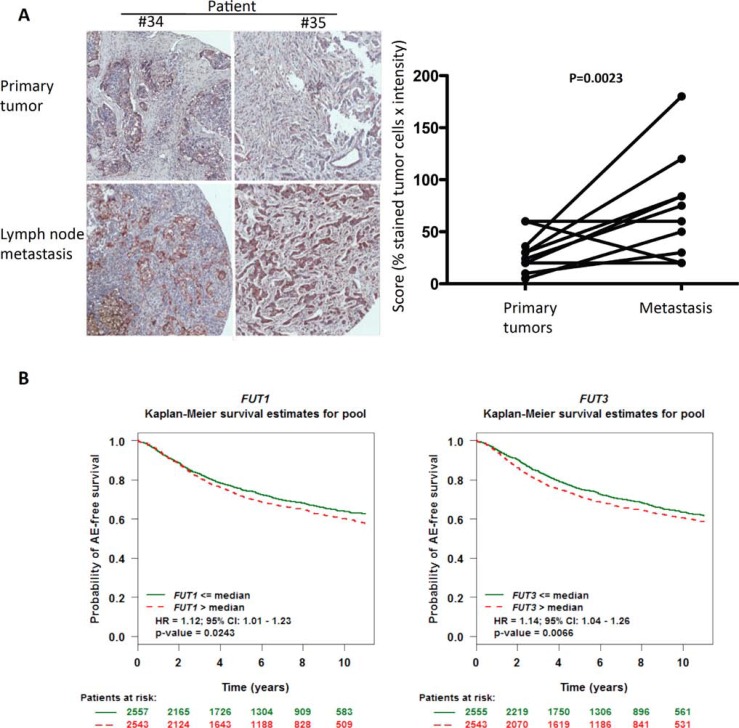
Associations between prolectin ligand expression, lymph node metastasis and clinical prognosis (**A**) The level of staining by the prolectin CRD was quantified on the 10 primary tumor/lymph node metastasis pairs from the breast TMA of Figure 4. A global score representing the proportion of stained tumor cells corrected by the staining intensity was established. Prolectin staining intensity was scored as +, +/++, +/+++ and ++/+++ and percentage of positive tumor cells was adjusted using coefficients of 1, 1.2, 1.5, 1.5 and 2 respectively. Pictures of two of the pairs are shown as examples. Statistical significance has been determined using a 2-tailed Wilcoxon test. (**B**) Computational analysis of breast cancer patient data were performed using the online platform bc genexminer (http://bcgenex.centregauducheau.fr). *FUT1* and *FUT3* expression are grouped into high and low level expression by comparison with the median expression of each gene. Analysis shown was done on all the patients irrespective of their nodal or estrogen receptor status (*AE: Any Event; HR: Hazard Ratio)*.

Since endogenous prolectin is expressed in lymph nodes, it has the possibility to interact with infiltrating tumor cells. In this case, it would be expected that the tumor cells retained in the lymph node are those that express more prolectin ligands. We therefore compared staining of paired primary breast tumors and lymph node metastasis with prolectin CRD. A score was established corresponding to the estimated percentage of labeled tumor cells, modulated by a color intensity index. The score was found to be significantly higher for the metastatic tissue than for the primary tumor tissue (Figure 6A).

We next wondered whether there could be some relationship between levels of *FUT1* and/or *FUT3* expression and the disease progression. We used the meta analysis tool bc-GenExMiner which compiles transcriptomic data from breast cancer studies (http://bcgenex.centregauducheau.fr/; [24]). Forest prognostic analysis was performed on the whole set of patients (irrespective of their nodal or estrogen receptor status) and the first pejorative event considered was represented by any relapse or death (referred to as “any event”). High-level expression (i.e. above the median expression of the gene) of both *FUT1* and *FUT3* was correlated to a bad prognosis with hazard ratios of 1.12 (*P* = 0.0243) and 1.14 (*P* = 0.00966) respectively (Figure 6B an Figure S13A). No such correlation was found for other fucosyltransferases. Similar results were obtained with the KM plotter online platform (http://kmplot.com/analysis/index.php?p=service&cancer=breast; [25]; See Figure S13B)

Collectively, these results suggest that prolectin could help implantation of metastasis in the lymph nodes through binding of fucosylated ligands on incoming tumor cells.

## DISCUSSION

In this study, we have shown that fucosylated motifs such as Le^y^ tend to disappear during the course of EMT and that this change reflects repression of the FUT1 and FUT3 enzymes expression in mesenchymal cells. Consistent with this change in glycosylation, we found that the bacterial fucose-binding lectin BC2L-C-Nt differentially stains epithelial and mesenchymal cells. This property was not shared by the well-characterized fucose-binding lectin UEA-I. *In vitro*, BC2L-C-Nt binds efficiently to H type 1/3, but poorly to H type 2 [13]. This means that if type 2 rather than type 1 structures are expressed, which seems to be the case in the breast cell lines, BC2L-C-Nt will poorly bind in conditions where the level of α1, 3/4 fucosylation is low. In contrast, UEA-I has a strong preference for H type 2 and will still bind, providing the cells have α1, 2 fucosyltransferase activity. This probably explains why BC2L-C-Nt display higher specificity than UEA-I for epithelial versus mesenchymal cells. We propose that BC2L-C-Nt could be a good tool to sort out cells with an epithelial phenotype in a mixed population. In the past few years, a great deal of effort has been put in the development of various systems for sorting circulating cancer cells in the bloodstream (see [26] for a review). Considering the vast heterogeneity of cancer cells, it is essential to target different markers to achieve the best sensitivity and specificity. In this context, BC2L-C-Nt could be used in combination with other antibodies or lectins to improve sorting of the epithelial cancer cells from blood. BC2L-C-Nt has been recently described as a good probe for embryonic stem cells (ES) or induced pluripotent stem cells (iPS) identification [27, 28]. Interestingly, those types of pluripotent stem cells tend to express epithelial markers such as epithelial cell adhesion molecule (EPCAM) or E-Cadherin, which is in contrast with the so-called cancer stem cells that are associated with a mesenchymal phenotype [29]. It is thus possible that BC2L-C-Nt could be used to distinguish between different types of stem cells.

We observed that both *FUT1* and *FUT3* were down-regulated in the course of EMT. Potential binding sites for SNAIL and ZEB transcription factors are present in the *FUT1* and *FUT3* promoters similar to those contained in the E-cadherin promoter, which is known to be repressed by proteins from these families [22, 23, 30, 31]. *FUT3* seemed to be more sensitive to the EMT-related repression as it was drastically down-regulated both by expression of Kras(v12) and of SNAIL, whereas *FUT1* was much more sensitive to SNAIL than to Kras(v12). TGF-ß treatment also decreased *FUT3* expression but not that of *FUT1*. Very recent data show that Kras induces EMT in breast cells *via* SLUG (*SNAI2)* rather than SNAIL (*SNAI1*) or ZEB [32], which might explain the difference for *FUT1* repression between MCF10A-Kras or MCF10A cells treated with TGF-ß on the one hand, and MCF10A-SNAIL on the other hand.

TGF-ß treatment was poorly efficient at inducing EMT in our hands. Using MCF10A cells, Zhang et al. have shown that TGF-ß-induced EMT is a two-step process, going through a SNAIL-driven intermediary stage before reaching the complete mesenchymal state [33]. For reasons difficult to explain, we could only achieve a partial transition. However, we think our results with cancer cell lines and with the two MCF10A-based EMT models clearly demonstrate that fucosylation is down regulated in breast mesenchymal cells.

Interestingly, Sakuma et al. have shown in colon cancer cells that following induction of EMT by EGF, *FUT2* expression is repressed *via* down-regulation of the colon-specific transcription factor CDX-2. However, in contrast to our results, the level of *FUT3* expression was up-regulated rather than down-regulated due to increased recruitment of c-Myc to the *FUT3* promoter [34]. It is possible that in the context of colon cells, a higher level of activating signals makes it possible to bypass the EMT-driven repression of *FUT3*. In a very recent study, Tan *et al.* have used a murine mammary cell line (NMuMG) with or without TGF-ß treatment to extensively analyze the glycosylation changes occurring during EMT. In a lectin microarray, they found reduced staining by the Fuc (α1, 3)-specific lectin from *Lotus tetragonolobus* on the TGF-ß treated cells. Transcriptome analysis also showed reduced expression of several FUTs [35].

Mesenchymal cells have a higher invasive potential than epithelial ones. Cancer cells invasiveness is often linked to increased expression of selectin ligands (SLe^x^ and SLe^a^) that allow extravasation and foreign tissues colonization [36–40]. It has been shown that increased expression of FUT1 in HT-29/M3 increases α2-fucosylation of the type 2 (Galß1-4GlcNAc-R) disaccharide which, by competing with sialylation, leads to a decrease of the synthesis of SLe^x^ and hence of the invasive and metastatic capacities of the cells [41]. In the study of Sakuma et al. [34], EMT-associated down-regulation of *FUT2* and induction of *FUT3* expression leads to increased levels of SLe^x^ on the cell surface. Thus, it seems contradictory that in breast cancer cell lines, induction of EMT leads to repression of *FUT3* expression and suppression of both neutral and sialylated Lewis antigens presentation. As SLe^x^ was detected on mesenchymal cells such as BT-549, MCF10A-KRAS or MCF10A-SNAIL with the KM-93 antibody only but not with the HECA-452 antibody, it is likely that this is due to the cross-reactivity of KM-93 with sialylated lactosamine [17], suggesting that the decrease of FUT3 expression also leads to a decrease of SLe^x^ presentation. Previous reports have indeed suggested that expression of SLe^x^ and E-Selectin ligands was dependant on FUT3/FUT6 expression in breast tumors and cell lines [3, 42].

Functional significance of Le^y^ overexpression by epithelial cancer cell is less well understood. *Hotta et al.* reported that Le^y^ was deleted in the invasive front of oral squamous tumors and that expression of FUT1 and Le^y^ in squamous cancer cell lines is associated with reduced proliferation and invasiveness [43]. However, in oral cancers, Le^y^ has also been suggested to promote cell migration *via* stabilization of EGFR and downstream signaling [44]. Interestingly, a recent study shows that overexpression of FUT1 in bladder cancer cell lines increases integrin ß1 activity and enhances cell adhesion [45]. Further studies are needed to better understand the regulation and functional consequences of glycosylation changes during EMT.

We also showed that the C-type lectin prolectin, expressed in lymph nodes, was a fucose-dependent epithelial cell adhesion molecule. Prolectin also reacts with polymannosylated ligands and therefore does not distinguish between epithelial and mesenchymal cells as well as BC2L-C-Nt when used as a probe for flow cytometry. Nevertheless, we showed that fucosylated cells could adhere to a layer of prolectin in low flow conditions. Mesenchymal or “de-fucosylated” epithelial cells show dramatically reduced adhesion to prolectin. Rat fibroblasts stably expressing Prolectin also showed reduced velocities when placed under flow on a tumor cell monolayer. We could not observe actual adhesion of the prolectin-expressing cells on tumor cells in those conditions. It might be argued that rat fibroblasts are not the most appropriate model to study cell-cell adhesion to human tumor cells. It is also possible that the density of prolectin at the fibroblast surface is not sufficient to allow firm adhesion such as what occurs on the surface coated with prolectin CRD. It would be interesting in the future to obtain a B-cell line with or without prolectin to perform the fluidic experiments in more physiological conditions. In addition, as prolectin has a cytoplasmic tail containing SH2 and SH3 domains [16], it would be interesting to determine if interaction of prolectin expressing B-cells with tumor cells triggers some signaling pathways in the B-cells and the consequences that such signaling could have in terms of B-cell activation and functional properties.

Although the exact role of prolectin interaction with tumor cells is not clear yet, we provide indirect evidence that it could be involved in metastasis implantation in lymph nodes. Firstly, meta-analysis of breast cancer data shows that high levels of expression of *FUT1* and *FUT3* are associated with a bad prognosis. A higher expression of genes coding those fucosyltransferases can lead to a higher presentation of fucosylated ligands for prolectin at the tumor cell surface. Secondly, when probing with the prolectin CRD, we observed more intense staining of lymph node metastasis than of the matching primary tumors, suggesting that tumor cells that have a greater ability to interact with prolectin, might initiate metastasis development more efficiently in the lymph nodes. It has to be noticed that the role of tumor cell interaction with prolectin-expressing cells will be difficult to directly test *in vivo*, since rodents do not possess an ortholog for the *CLEC17A* gene.

**Figure 7:**
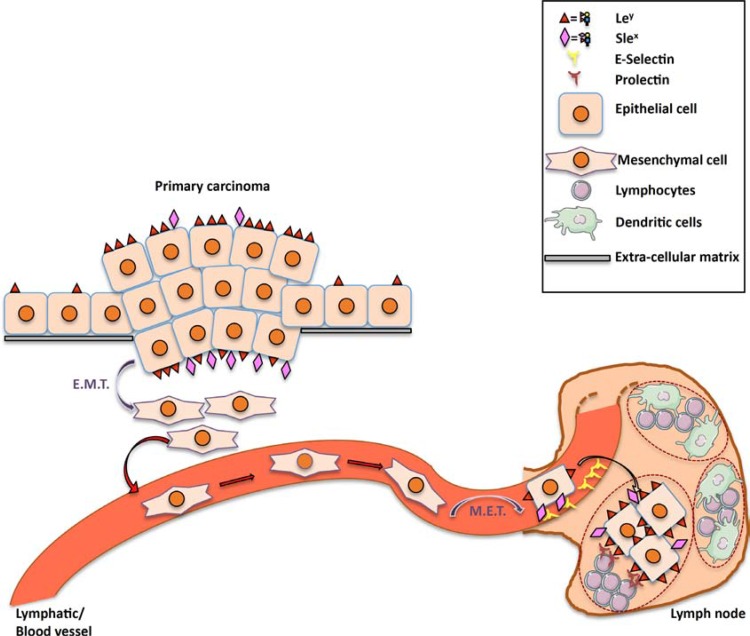
Proposed model for the roles of prolectin and fucosylated antigens in lymph node metastasis Increased expression of both neutral fucosylated glycan motifs such as Le^y^ and charged fucosylated structures such SLe^a/x^ is observed on primary tumor cells as compared to their normal counterpart. These fucosylated markers, are lost during EMT that allows tumor cells to migrate and pass into the blood or lymphatic streams. When reaching the target lymph node, these cells use the selectin ligands for extravasation and undergo MET. The latter step will be accompanied by re-expression of fucosylated ligands through FUT1 and FUT3 expression, thereby allowing binding to prolectin that will contribute to the settlement of the metastatic cells in the lymph node.

We propose the model illustrated in Figure 7. In a carcinoma overexpressing fucosylated motifs such as Le^y^ and SLe^x^, some cells will go through an EMT, allowing them to detach from their original tissue and migrate to blood or lymphatic vessels. During EMT, Le^y^ and SLe^x^ disappear from the cell surface. By a process of MET, tumor cells will re-express fucoslylated ligands. The SLe^x^ motifs allow cancer cells to interact with the endothelium of the lymph node afferent vessels and to exit from the blood/lymph stream. Upon entrance into the lymph node, neutral fucosylated ligands allow carcinoma cells to interact with germinal center B cells expressing prolectin, which will help them to settle in the lymph node.

Reticker-Flynn and Bhatia have just described a somewhat similar involvement of a lectin-carbohydrate interaction in the establishment of the metastatic niche. In their case, overexpression of the Thomsen-Friedenreich antigen (Galß1-3GalNAc) by lung adenocarcinoma metastatic cells allowed them to interact with galectin-3 expressed on pro-tumorigenic leucocytes [46].

Interestingly, during the preparation of this manuscript, two studies have challenged the idea that EMT would be required for metastasis [47, 48]. This means that epithelial cancer cells could actually leave the primary tumor, migrate to the lymph node and interact with prolectin without the need of going through EMT/MET cycles.

In conclusion, our study provides a direct link between the mesenchymal state and repression of some fucosyltransferases expression. We also describe the bacterial lectin BC2L-C-Nt as a new tool to monitor EMT and the human C-type lectin prolectin as a potential new player in lymph node metastasis.

## MATERIALS AND METHODS

### Cell culture

MCF-7, T47-D, BT-549, DU-145 and OVCAR3 cells (obtained from ATCC) were grown in RPMI 1640 GIBCO (Life Technologies) supplemented with 10% Fetal Calf Serum (FCS) GIBCO. The CHO cell lines were grown in RPMI-10% FCS supplemented with 10 μg/ml each of adenosine, thymidine and 2-deoxy-adenosine (Sigma). CHO-H cells were obtained by stable transfection of CHO cells with the rabbit *FUT1* enzyme as described in [49]. CHO-Le^a^ and CHO-Le^b^ were a kind gift from Jan Holgersson [50]. CAL-51 (obtained from DSZM), MDA-MB-231, ZR-75.1, HT-29 (obtained from ATCC), RAT6-Neo and RAT6-CTLY11 were grown in DMEM containing 4,5 g/l glucose and supplemented with 5% (CAL51, MDA-MB-231) or 10% FCS. 100 μg/ml of G418 (neomycin) at 100 μg/ml was added to the RAT6 fibroblasts medium to maintain prolectin expression [16]. MCF10A-Kras(v12) and the empty vector control MCF10A-LXSN are a kind gift from Dr Ben Ho Park [51]. MCF10A-SNAIL cells and their corresponding MCF10A-Puro^R^ controls were obtained from Dr Stéphane Ansieau [52]. MCF10A cell lines were grown in DMEM:HamF12 (1:1) GIBCO supplemented with 5% Horse Serum GIBCO, 0,5 μg/ml hydrocortisone (Sigma), 20 ng/ml human epidermal growth factor (Life Technologies), 10 μg/ml bovine insulin (Sigma), 100 ng/ml Toxin from *Vibrio cholerae* (Sigma) and 50 mM HEPES GIBCO. All culture media were supplemented with 2 mM L-glutamine GIBCO and 1% penicillin-streptomycin GIBCO^®^. For MC10A-SNAIL and their MCF10A-Puro^R^ counterpart, 0.5 μg/ml of Puromycin was also added to maintain the plasmid. Cells were grown at 37°C with 5% CO_2_.

### Treatment with TGFβ1 and TGFβ signaling inhibitor

To induce EMT, cells were treated with 5–10 ng/ml of TGF-ß1 (e-Bioscience or R & D). Cells were passaged continuously under TGF treatment or treated during 4 days, changing the medium every two days. In some experiments, cells were treated with 0,5 μM of the TGFßRI inhibitor A83-01 (Sigma Aldrich) to maintain the epithelial phenotype (control cells) or to improve the MET process after TGF-ß withdrawal.

### Antibodies and lectins

We used the following antibodies to detect glycoantigens: anti-Le^a^ (7-LE; 1/100); anti-Le^b^ (LM- 137; 1/10), anti-Le^x^ (3E1; 1/10; EFS Nantes), anti-Le^y^ (12- 4LE; 1/400), anti-H type 3 (Mbr1; 5 μg/ml; Enzo life Science), anti-SLe^x^ (KM-93; 10 μg/ml; Merck-Millipore, Notthingham, U.K and HECA-452; 250 μg/ml; BD Bioscience) and anti-SLe^a^ (19-9; 1/200). 7-LE, LM-137, 12-4LE and 19-9 antibodies were from J. Bara (Villejuif) and U892 (Nantes). All the above antibodies are mouse monoclonal antibodies except from HECA-452, which was produced in rat. E-Cadherin was detected with 24E10 rabbit monoclonal antibody (1/200; Cell Signaling Technology) and vimentin with IF01 mouse monoclonal antibody (1/20 for flow cytometry and 1/50 for immunofluorescence; Calbiochem, Merck Millipore). Prolectin was detected with a rabbit polyclonal antibody [16].

We also used several recombinant bacterial lectins (BC2L-A, BC2L-C-Nt, PA-IL, RSL) produced in *E. Coli* in the CERMAV laboratory (CNRS UPR5301, Grenoble, France) as well as the plant lectin UEA-I (Vector laboratories, Burlingame, CA).

### Plasmid construction

For expression of the CRD from prolectin, a pET-24a-Pro vector was obtained by gene synthesis from GENEART (Life Technologies; Regensburg, Germany). This plasmid drives expression of the CRD of prolectin (residues 247 to 380) with the biotinylation acceptor sequence GLNDIFGAQKIEWHE appended at the C-terminus, as described in [16].

### Preparation of biotinylated prolectin tetramers

A 3 Liters culture of *E.Coli* strain BL21(D3) transformed with the pET-24a-Pro plasmid was prepared and prolectin expression was induced by addition of 1 mM Isopropyl ß-D-1 thiogalactopyranoside (IPTG). After incubation for 5 h at 37°C, bacteria were pelleted and resuspended in saccharose buffer before freezing. Bacteria were thawed, sonicated and digested with 300 μg/ml Lyzosyme. Inclusion bodies were washed 8 times with wash buffer (50 mM Tris-HCl pH 8, 100 mM NaCl, 0.5% Triton X-100 + protease inhibitors without EDTA (Roche)), resuspended in 8 M urea for protein denaturation, collected by ultracentrifugation and protein concentration was determined. Inclusion bodies (8– 16 mg) were resuspended in 125 ml of renaturation buffer (100 mM Tris-HCl pH 8, 25 mM CaCl2, 0.5 M NDSB256 (Sigma), 5 mM reduced glutathione, 0.5 mM oxydized glutathione + protease inhibitors without EDTA (Roche)) and incubated for 3 days at 4°C under slow agitation. The renatured protein was concentrated on filters with 1-3 kDa cut-off (Amicon) and biotinylated by incubation with the BirA enzyme for 5 h. The biotinylated protein was applied on a HiLoad S200 gel filtration column (GE Healthcare). Elution was performed in phosphate buffer saline (PBS) and fractions corresponding to the monomer of prolectin were pooled, aliquoted and stored at −80°C.

Since lectins affinity for glycosylated ligands is generally improved when they are in multimeric states, we formed tetramers of prolectin by taking advantage of the property of the streptavidin molecule to have 4 biotin-binding sites. Biotinylated-prolectin monomers were incubated with streptavidin-horseradish peroxidase (strepatvidin-HRP; Vector laboratories, Burlingame, CA) or streptavidin-phycoerythrin (streptavidin-PE; BD Pharmingen) at a molar ratio of 1:5 in TBS-BSA, 10 mM CaCl_2_ for 1–2 h at room temperature. Tris-buffered saline (TBS) was used instead of PBS to avoid the formation of calcium phosphate precipitates. These tetramers were then used in the different assays described below.

### Enzyme linked lectin assay

Oligosaccharides conjugated to polyacrylamide beads (PAA) were obtained from N. Bovin (Shemyakin Institute of Bioorganic Chemistry, Moscow, Russia). Human serum albumin (HSA) conjugated with oligosaccharides were purchased from Isosep (Tullinge, Sweden). Detailed formulas of the oligosaccharides are given in Table S3.

Synthetic oligosaccharides were used at 0.1 mg/ml. All samples were diluted in coating buffer (Carbonate-Bicarbonate 0.1 M pH 9.5) and adsorbed in duplicates on “Maxisorb” 96 well- plates (Nunc, ThermoFisher Scientific, Waltman, MA) with overnight incubation at 37°C. After 3 washes with PBS-0.05% Tween20 (PBS-T), blocking of the non-specific protein binding sites was performed with PBS-5% BSA for 1 h at 37°C. Biotinylated BC2L-C-Nt diluted to 1 μg/ml in TBS-1% BSA, 10 mM CaCl_2_ was added to the plate for 2 h at room temperature. After 3 washes with PBS-T, streptavidin-HRP at 5 μg/ml in PBS-1% BSA (in the wells incubated with BC2L-C- Nt) or 4 μg/ml of prolectin tetramerized with streptavidin-HRP in TBS-1% BSA, 10 mM CaCl_2_ (see above) were added to the plate, followed by 2 h incubation at room temperature. After 3 washes with PBS-T, plates were developed by adding 200 μl/well of OptEIA substrate (3, 3′, 5, 5′ tetramethylbenzidine, BD). The reaction was stopped by adding 50 μl/well of 1 M phosphoric acid and optical density was read on a iMark Microplate Reader (Bio-Rad).

### Flow cytometry

Cells were trypsinized, aliquoted and resuspended in PBS-0.1% BSA (PBS-BSA). After 1–2 h incubation at 4°C with biotinylated lectins diluted in PBS-BSA, cells were washed 3 times with PBS-BSA and incubated with streptavidin-PE (BD Pharmingen) for 30 mn at 4°C. Alternatively cells were incubated directly with 10 μg/ml prolectin in complex with streptavidin-PE in TBS-0.1% BSA, 10 mM CaCl2 (or 2.5 mM EDTA for the negative control) for 1–2 h at 4°C.

For detection of the glycosylated antigens, cells were incubated with primary antibodies diluted in PBS-BSA for at least 30 mn at 4°C. After washes with PBS-BSA, the cells were incubated with Goat F(ab’)_2_ anti- mouse IgG (H + L)-FITC (Beckman) or Goat anti-rat IgG (H + L)-FITC (Novex, Life Technologies) 1/200 in PBS-BSA for 30 mn at 4°C.

In all cases, cells were washed 3 times with PBS, resuspended in PBS and analyzed on a FACSCalibur flow cytometer (BD).

### Immunofluorescence

Cells were fixed with paraformaldehyde 4% in PBS for 20 mn at room temperature. Blocking and permeabilization were then performed simultaneously using PBS-0.1% Triton X-100-10% FCS. Cells were incubated sequentially with anti-E-Cadherin and anti-Vimentin (see above) diluted in PBS-10% FCS for 1 h at room temperature. After washes with PBS-10% FCS, cells were incubated with Goat anti-rabbit IgG (H + L)-DyLight 488 (KPL), 1/400 and then with Goat anti-mouse IgG (H + L)-Rhodamin (Chemicon, Merck Millipore), 1/200 and NucBlue Live Stain (Molecular Probes, Life Technologies). After washes with PBS, slides were mounted in ProLong Gold anti-fade reagent (Molecular Probes, Life Technologies).

### Tissue sections

Ethanol-fixed colorectal tumor sections were obtained after cancer surgery. They were collected before the French law 88-138 of December 20, 1988 concerning resection of human tissues after death for scientific investigations. The samples were obtained from the Nantes University Hospital Center for Biological Resources (http://relib.fr), under the Cancerology program approved by the ministry of research (approval DC-2011-1399). A breast tumor tissue microarray (TMA; formalin-fixed; 40 tumors, 10 paired lymph node metastasis and 10 paired adjacent “normal” tissue), a lung tumor TMA (formalin-fixed; 40 tumors, 7 paired lymph node metastasis, 2 paired bone metastasis, 1 paired soft tissue metastasis and 10 paired adjacent “normal” tissue) as well as a uterine cervix tumor TMA (formalin-fixed; 40 tumors, 5 paired lymph node metastasis and 5 paired adjacent “normal” tissue) were bought from SuperBiochips Laboratories Ltd (Seoul, South Korea). The company certifies that the “human material has been removed or collected with the donor's prior consent and that no payment whatsoever has been made to the latter”.

### Histochemistry

The tissue sections were deparaffinized, then endogenous peroxidases were blocked by 20 min incubation in PBS-0.3% Hydrogen peroxide. Non-specific binding was blocked by 60 min incubation in PBS-5% BSA. Prolectin-Streptavidin HRP tetramers in TBS-1% BSA-10 mM CaCl2 were added for 2 h at room temperature. After 3 washes with PBS, peroxidase substrate (AEC kit, Vector Laboratories) was added to the slide for 10–20 min followed by quick soaking in Mayer's hematoxyllin solution (Vector Laboratories) for contrast staining.

Slides were imaged with a Nanozoomer slide scanner (Hamamatsu, Hamamatsu City, Japan).

### Adhesion assay under flow

Microfluidic experiments were performed using the BioFlux system (Fluxion) in a 48-well plate format.

BioFlux^®^ microchannels were coated with tetramers of prolectin-streptavidin (100 μg/ml) in coating buffer (carbonate-bicarbonate 0.1 M pH 9.5) containing 0.5 mM CaCl2, overnight at 37°C. Cells were trypsinized, counted and resuspended at 5 × 10^6^ cells/ml in PBS containing Ca^2+^ and Mg^2+^ and 5% FCS. 25 mM of D (+)-Galactose (Sigma) or L-(−)-Fucose (Sigma) were added at final concentrations of 25 mM as indicated in Figure legends. The cell suspension was loaded in the entry well of the BioFlux plate and a shear flow of 0.05-0.1 Dyn/cm^2^ was applied by the BioFlux pump, to inject the cells into the microchannel. A Series of 600 pictures, corresponding to 60-second films were recorded with a Leica DMI 6000B microscope. After the video acquisition, microchannels were washed 10 min with PBS containing Ca^2+^, Mg^2+^ at 4 Dyn/cm^2^ in order to remove unbound cells and pictures of the cells attached along the whole channels were taken.

In some cases cells were detached by flushing with PBS-0.02% EDTA and trypsin and stained with prolectin for fluorescence or flow cytometry analysis. Video data were analyzed by chronophotography on 10 pictures segments, using the Fiji software (http://fiji.sc).

### qRT-PCR

RNA extractions were performed using the Nucleospin RNA II purification kit (Macherey Nagel) following instructions of the manufacturer. RNA concentration was measured using a Nanodrop (Thermo Fisher Scientific) instrument. Reverse transcription was performed on 0.5-1 μg of total RNA with oligo-dT and the Superscript II reverse transcriptase (Life Technologies) as recommended by the manufacturer with 1-2 μl of the cDNAs used for the qPCR step.

For *FUT1* and *FUT3*, the Taqman Universal PCR Mastermix (Applied Biosystems, Life Technologies) was used with the primers and probes described by Nyström et al. ([53], See Table S1). *GAPDH* was used for normalization using Taqman Pre-developped Assay Reagents primers and probe mix (Applied Biosystems, Life Technologies). For *CDH1* and *B-Actin*, qPCR was performed using the Brilliant II SyBrGreen Mastermix (Agilent) and primers indicated in Table S2. qPCRs reactions were ran on a Mx3500P qPCR machine (Agilent). For normalization of the qPCR results, the ΔΔCT method was applied.

## SUPPLEMENTARY MATERIALS FIGURES AND TABLES



## Abbreviations

HBGA: Histo-Blood Group Antigen
EMT: epithelial-to-mesenchymal transition
MET: mesenchymal-to-epithelial transition
Le^a^: Lewis A
Le^b^: Lewis B
Le^x^: Lewis X
Le^y^: Lewis Y
SLe^x^: Sialyl-Lewis X
SLe^a^: Sialyl-Lewis A
CSC: cancer stem cell
ES: embryonic stem cell
iPS: induced pluripotent stem cell
CRD: carbodhydrate recognition domain
FUT: fucosyltransferase
TGF-ß: transforming growth factor ß
HSA: human serum albumin
PAA: polyacrylamide
KFN: kifunensine
MFI: mean fluorescence intensity
CHO: Chinese hamster ovary
FCS: fetal calf serum
PBS: phosphate-buffered saline
TBS: Tris-buffered saline
HRP: horseradish peroxidase
TMA: tissue microarray
