# Supplementary material for: Carcinoma-associated fucosylated antigens are markers of the epithelial state and can contribute to cell adhesion through CLEC17A (Prolectin)
Source: Oncotarget. 2016 Feb 18;7(12):14064–82. doi: 10.18632/oncotarget.7476 (PMC4924698; doi:10.18632/oncotarget.7476)
Supplement: Supplementary file 1 [file oncotarget-07-14064-s001.pdf]

Carcinoma-associated fucosylated antigens are markers of the epithelial state and can contribute to cell adhesion through *CLEC17A* (Prolectin)

Supplementary Materials

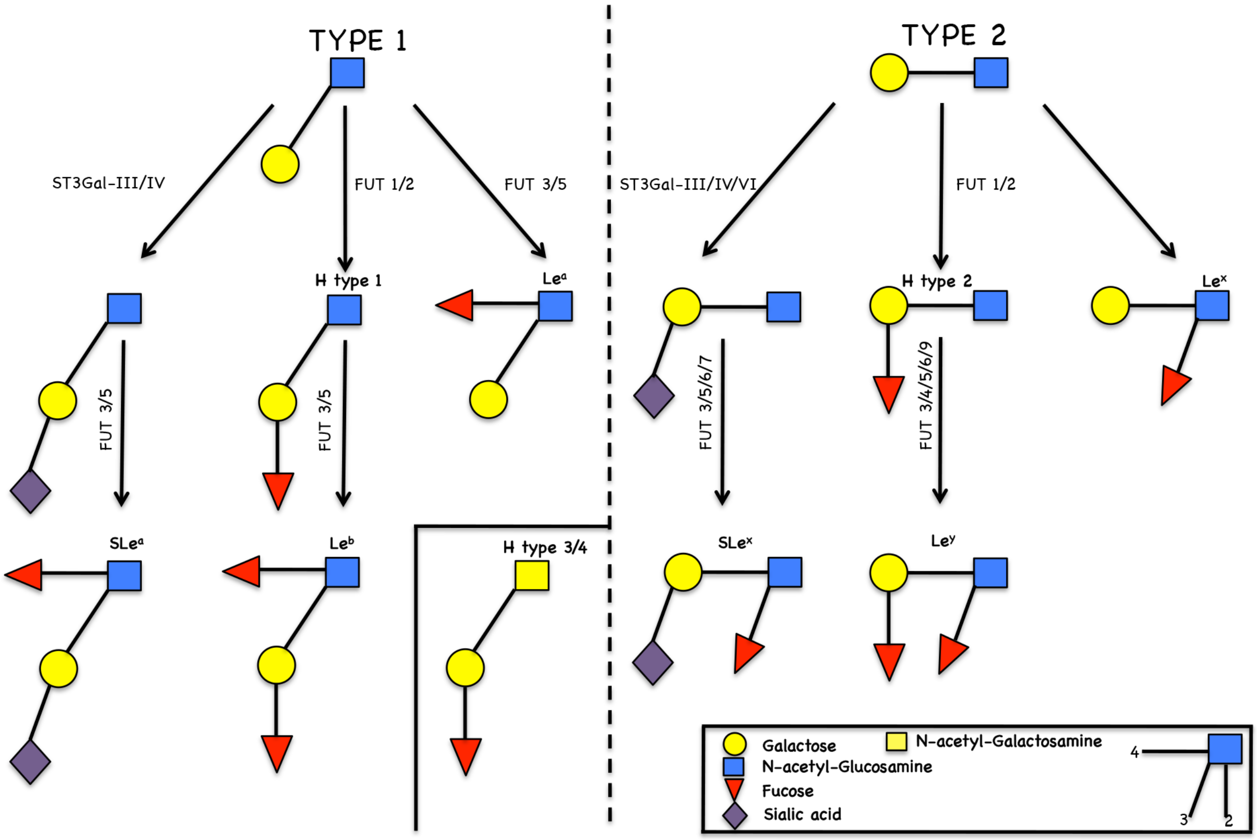

Supplementary Figure S1: Biosynthetic pathway of the histo-blood group antigens. α1, 2 fucosyltransferases (FUT), α1-3/4 FUT and α2-3 sialyltransferases (ST3Gal) mainly responsible for synthesis of the various intermediates are indicated. N-acetyl galactosamine or galactose moieties can be added to the terminal galactose to make the A or B antigens respectively.

A

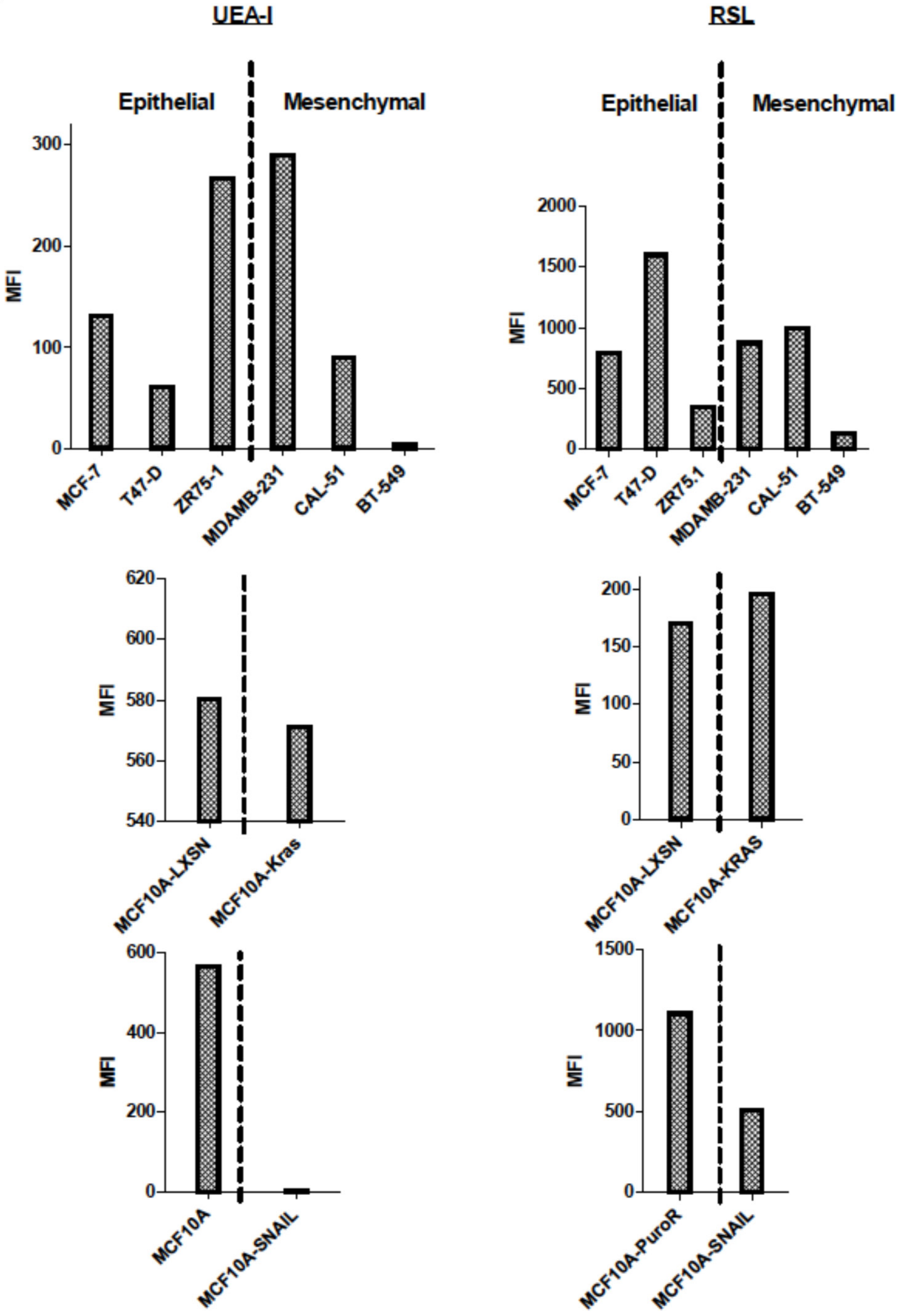
UEA-I
RSL
Epithelial
Mesenchymal
Epithelial
Mesenchymal
MFI
300
200
100
0
MCF-7
T47-D
ZR75-1
MDAMB-231
CAL-51
BT-549
2000
1500
1000
500
0
MCF-7
T47-D
ZR75.1
MDAMB-231
CAL-51
BT-549
620
600
580
560
540
MCF10A-LXSN
MCF10A-Kras
200
150
100
50
0
MCF10A-LXSN
MCF10A-KRAS
600
400
200
0
MCF10A
MCF10A-SNAIL
1500
1000
500
0
MCF10A-PuroR
MCF10A-SNAIL

B

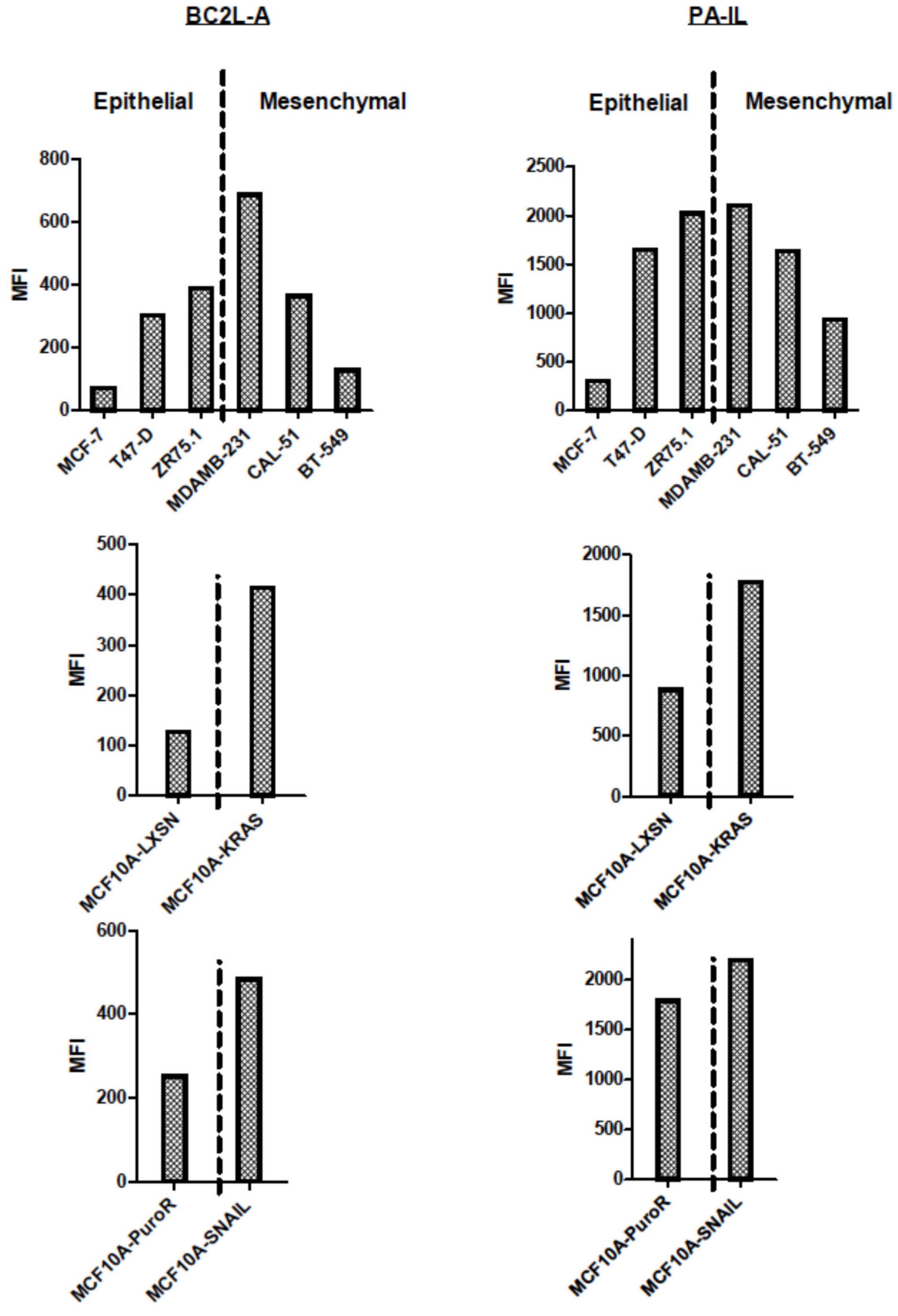

Supplementary Figure S2: Lectin binding on breast cell lines. The indicated breast cell lines have been stained with recombinant biotinylated lectins followed by streptavidin-PE and analysed by flow cytometry. Mean fluorescence intensities are presented. The results are representative of at least two experiments.

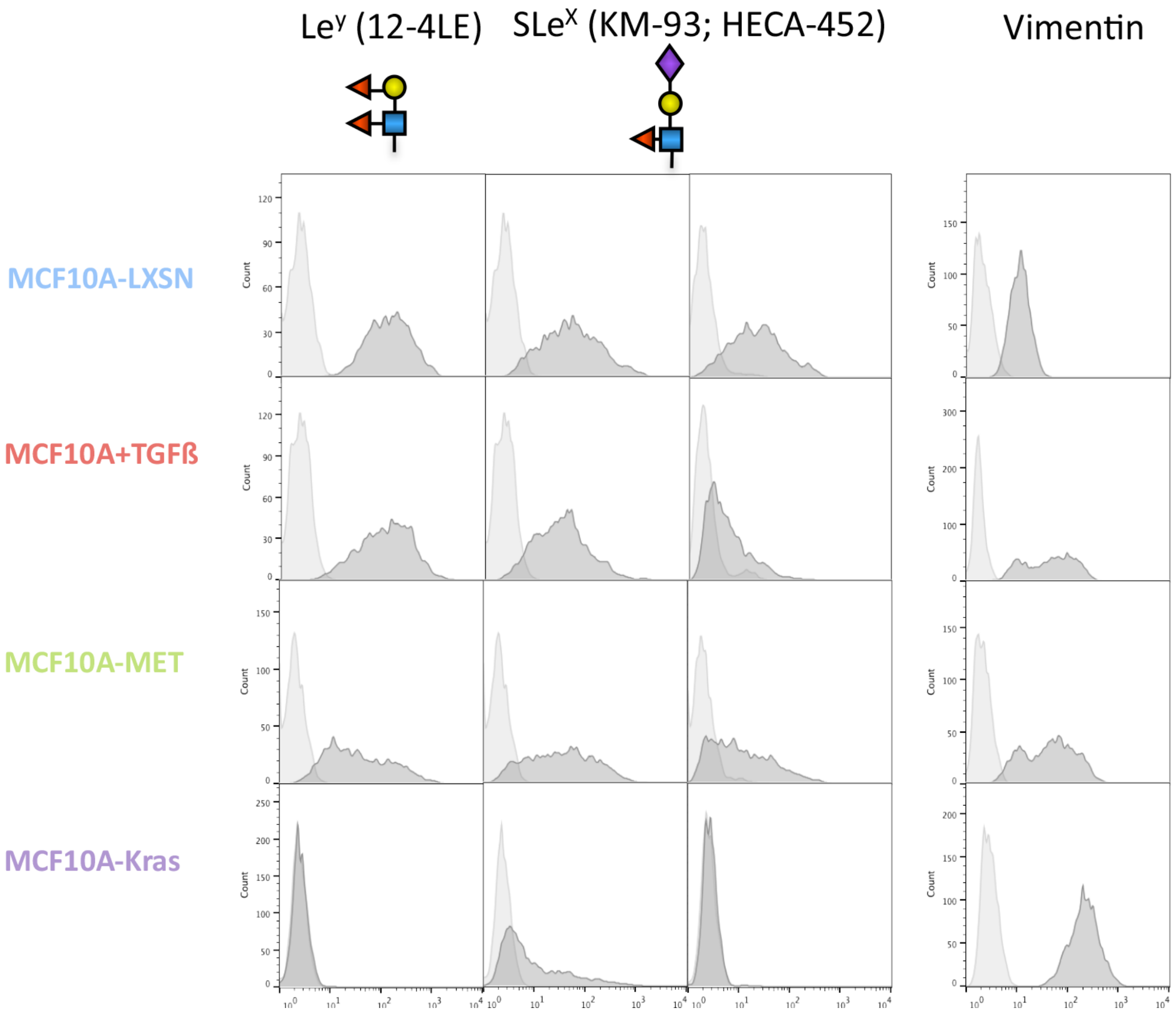

Supplementary Figure S3: Expression of fucosylated antigens by MCF10A cells treated with TGF-β. MCF10A-LXSN epithelial cells were treated with either 0,5 µM A83-01 (MCF10A-LXSN) or 5 ng/ml TGF-β R&D (MCF10A-TGF) during four days. TGF-β was then withdrawn and cells were grown four more days in presence of 0,5 µM A83-01 (MCF10A-MET). Cells were analyzed by flow cytometry as described in Figure l.

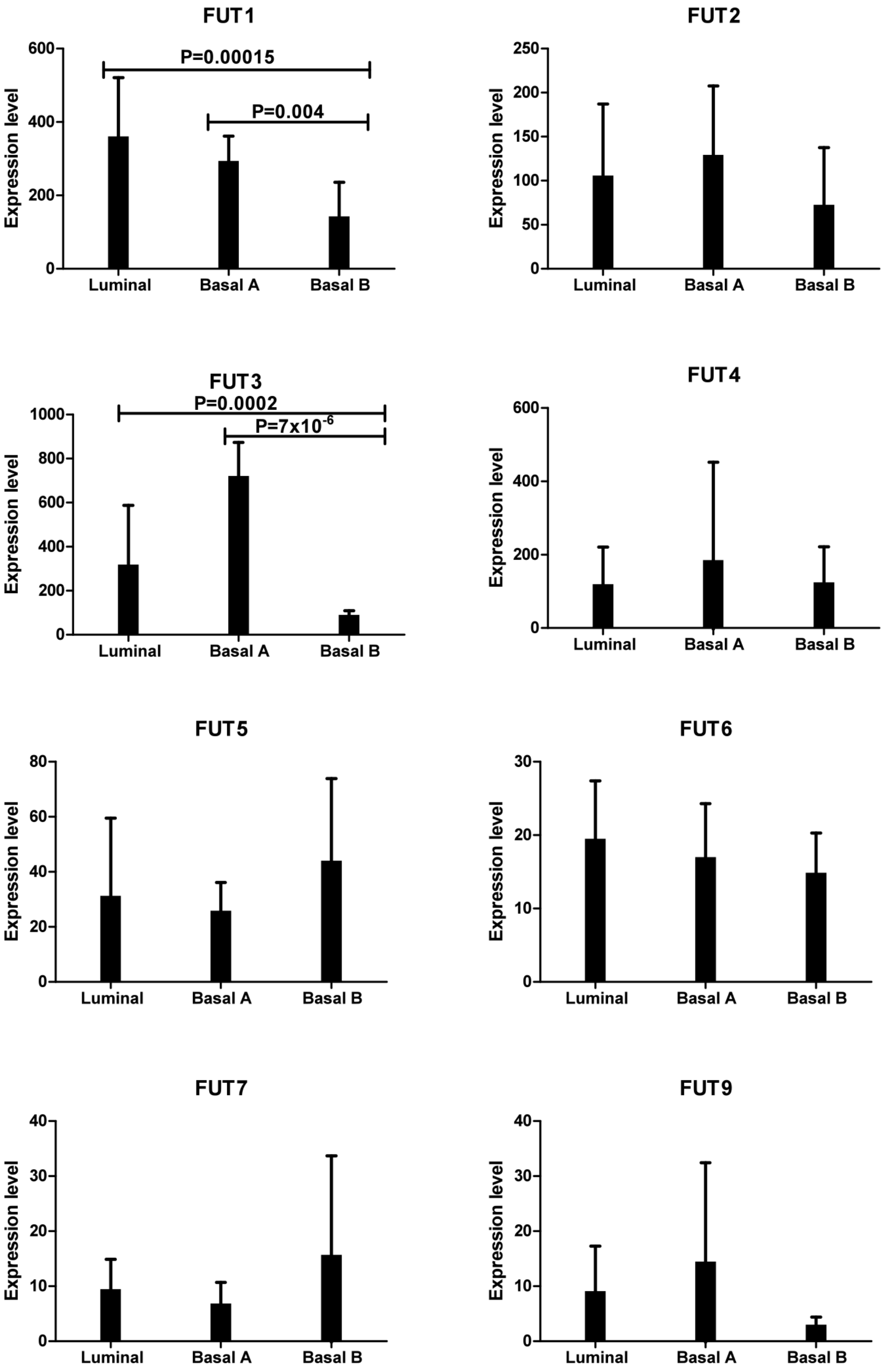

Supplementary Figure S4: Fucosyltransferases genes expression in breast cell lines. Expression levels of the FUTα1-2 (FUT1 and 2) and FUTα1-3/4 (FUT3, 4, 5, 6, 7 and 9) genes were extracted from a transcriptomic dataset of 51 breast cancer cell lines available in the NCBI database (GEO n° GSE12777) thanks to the online analysis tool BioGPS (http://www.biogps.org) and cell lines were sorted according to their overall gene profile subtypes (according to the classification of Neve RM et al, *Cancer Cell* 2006 and Kao J et al *PlosOne* 2009). Mean expression level for each *FUT* gene and standard deviations are shown.

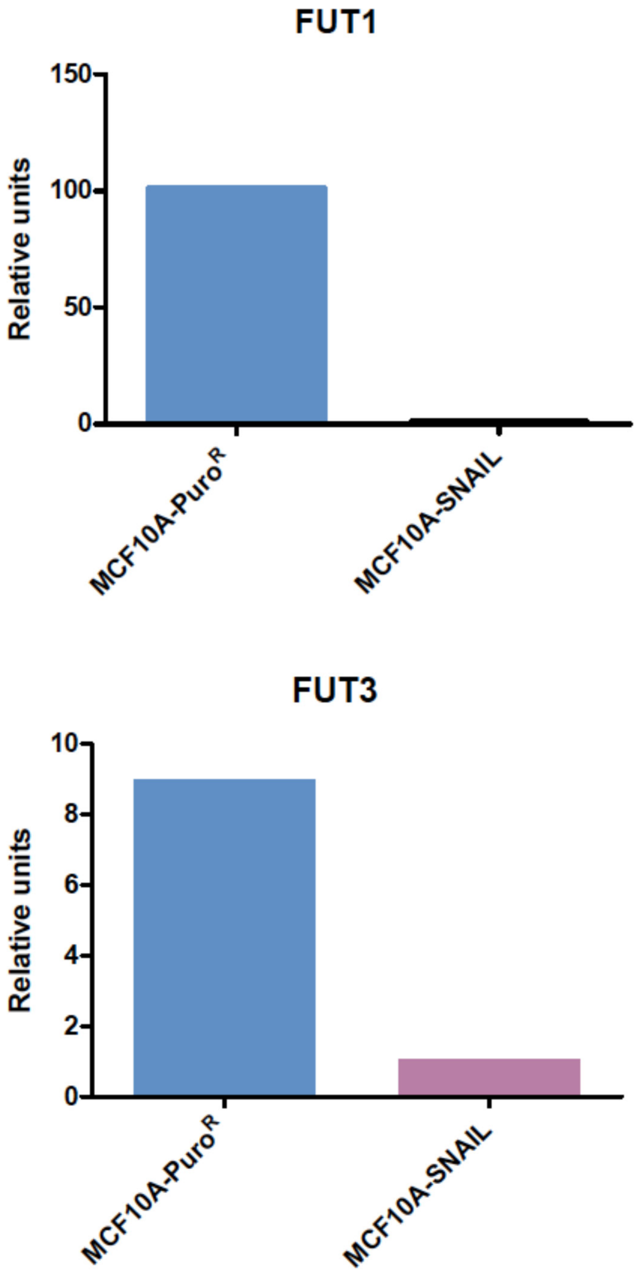

Supplementary Figure S5: Fucosyltransferases genes expression in MCF10A-Puro^R/MCF10A-SNAIL. Total RNAs were extracted from the different cell lines, cDNAs were then synthesized using oligo-dT and subjected to qPCR analysis with *FUT1*, *FUT3* and *GAPDH* specific primers and probes. *FUT1* and *FUT3* expression levels are presented relatively to *GAPDH* expression. Data shown are representative of three independent experiments.

A

-pFUT1

aaattaacttattattttttttgagacagagtcttgctgtactactcaggctggaggacagtggcacgatctcggctccctgcaacctc**cacct**c

ccaggttcaagtgattctgcctcagcccccccccgagtagctgggacaacagacgcccgccaccacgtctggctagtttttgtatttttagtag

agatggggtttcactgtgttggccaggctgctctaactcctgacctcaggcaatc**cacctg**cctcagcttcccaaagtgctgggattacaggc

gtaagccatggtgcctggtcttattttatttatttgtttacttatttatttattttgagactttttgagtcttgctctgtcgcccaggctggagtgcaa

tggctcgatctcagctcactgcaacctc**cacct**ccgggattcaagcgattctcctgcctcagcctcccaagtagctgggattacaggcatgtg

ctacca**cacctg**gctaatttttgtatt

-pFUT3

attacaaagtgcctccttaagggcagggggggaggatattacaaag**cacct**cctcaagggtgacgagggtatattgtacaaagtatgttcac

aagggcgggggaatatcacaaagtacattattgcaagggggtggtgtattgtcacaaagtcaattgatcagttagggtgaggcaggaacaa

atcgcaatggtggaatgtcatcagttaaggcaggaactggctattttcactttttttctggatcttcagttgcttcaggccatctggatgtacacg

tgcaggtcacaggggagatggtggcatagcttgggctcagaggcctgaagaattgatcccttacaaaagtctagtattgtctgattcacccac

ttggtccctggactctccctgggtctggtatgcaat**aggtg**cataagcaatgtttgttgattgactgactgaatgggtgggatggttaaaattct

gttcacttccctccctttaccatctcccgagaaacccatggggcccccgtgggacccactttgagaatcaaagctttagaccagcgttttcactt

tatggatggaccactgtgcacccaaagccaggcagccttcaggctacagatggagcccgggtcacgtcccgggcctccaagtccctgcacc

cttatccggtctcacaggcgagattaggacaccccggaaactggcttccagacaatatccctgctgcaggggagaacaccctaggt**cacct**

ggtga**caggtg**tgtgctgcaatgtacacgtacttgttcctccccttctct

B

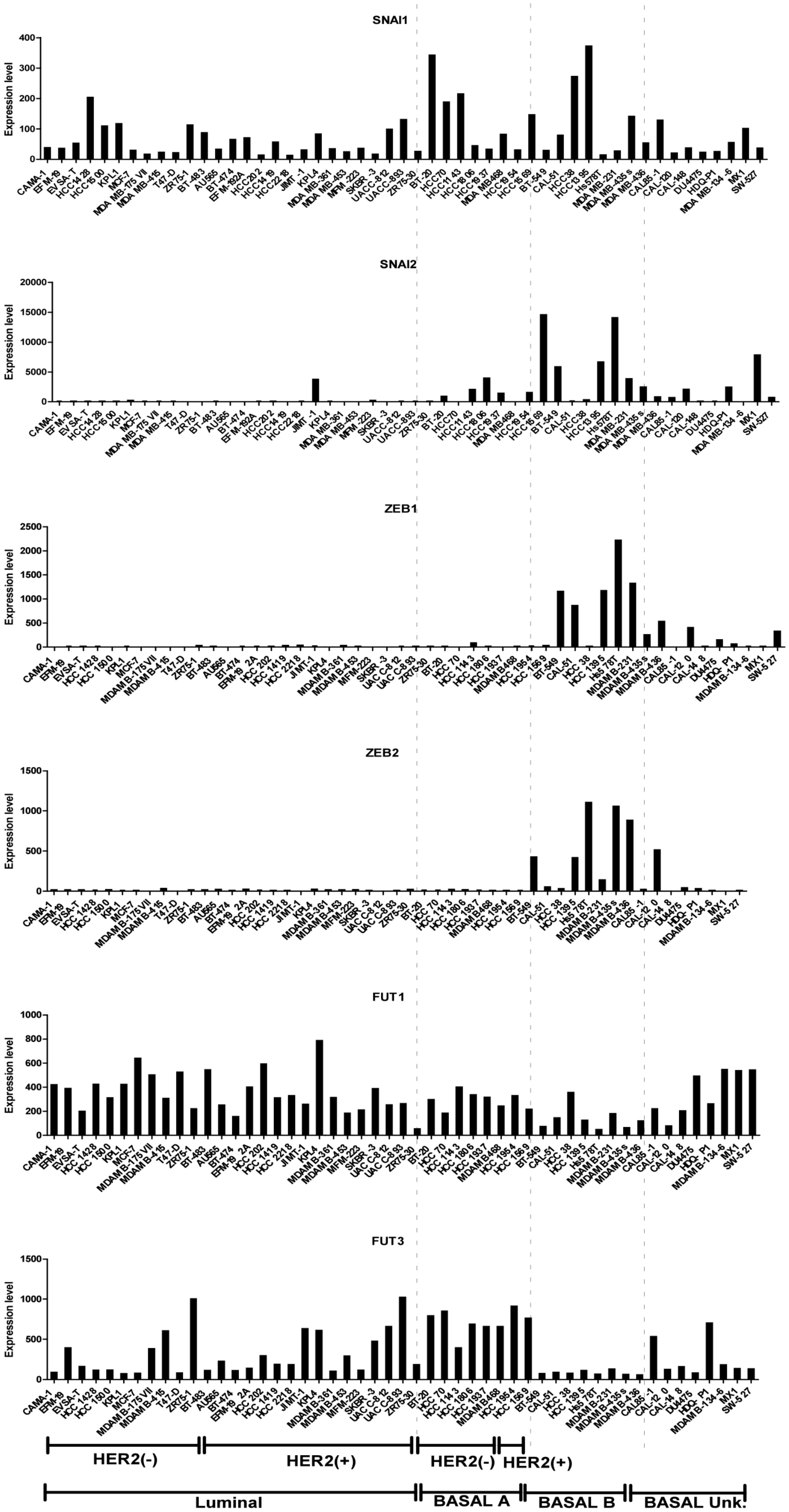

Supplementary Figure S6: *FUT1* and *FUT3* promoters contain binding sites for EMT-inducing transcription factors. (**A**) Sequences of *FUT1* and *FUT3* promoters. The E2-boxes CACCTG or CAGGTG that are binding sites for SNAIL are underlined. Moreover, bipartite elements (C)AGGTG/CACCT(G) (in red) are binding motifs for ZEB1/2. (**B**) Expression of *FUT1* and *FUT3* varies inversely with that of *ZEB1/2*. Data of the 51 breast cancer cell lines transcriptome were analysed as described above. (*Basal Unk*.: Basal cell lines for which the A or B classification is unknown, *SNAI1* = SNAIL; *SNAI2* = SLUG).

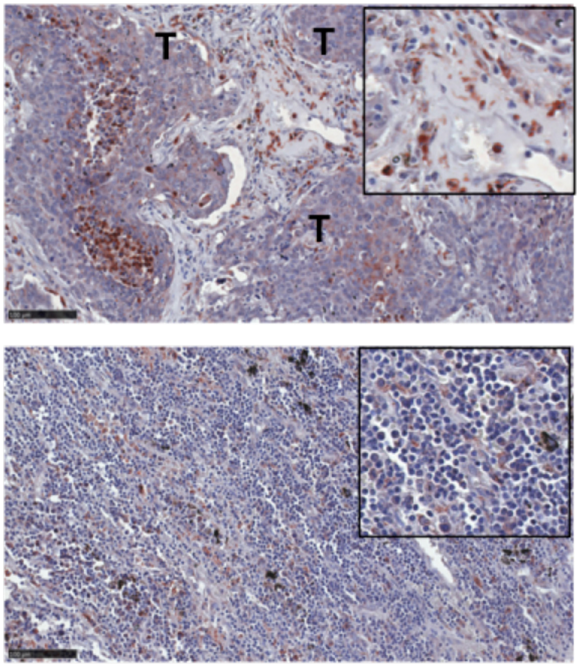

Supplementary Figure S7: Staining by prolectin of leucocyte populations infiltrated in tumors. Tetramers of biot-prolectin CRD/streptavidin-HRP were bound to a paraffin embedded TMA from lung cancer. An example of tumor is shown with infiltrating leucocytes strongly stained by prolectin as well lymphoid tissue from the same patient. 20x magnifications are shown with inlets at 40x magnfication. (T = Tumor).

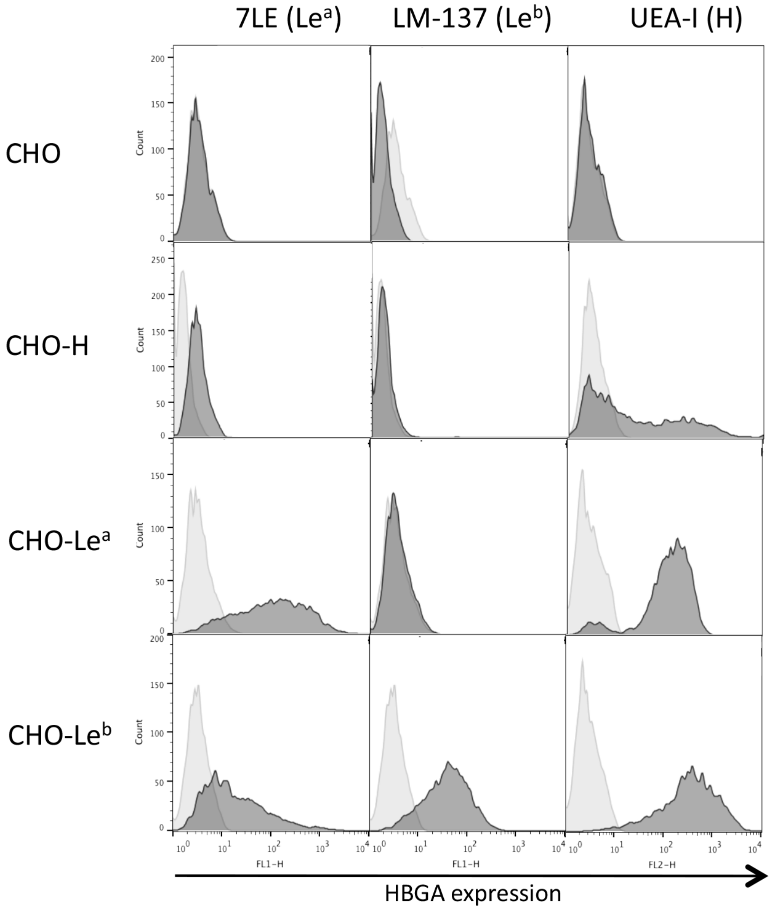

Supplementary Figure S8: Phenotyping of the CHO, CHO-H, CHO-Le^{a} and CHO-Le^{b}. The CHO cell lines were analysed by flow cytometry with the antibodies 7LE (anti-Le^{a}) and LM137 (anti-H type 1/Le^{b}) followed by anti-mouse-FITC and the biotinylated lectin UEA-I (α2-Fucose specific) followed by streptavidin-PE.

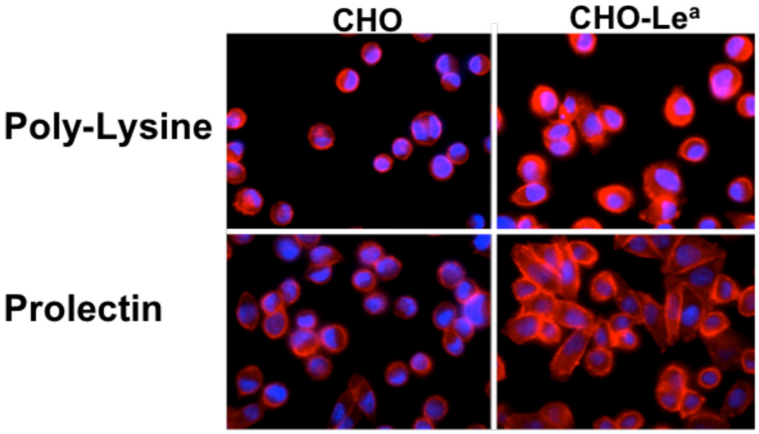

Supplementary Figure S9: Comparison of CHO-Le^a binding to poly-lysine and a prolectin coating. Lab-tek chamber slides have been coated with prolectin/strepatvidin tetramers or poly-L lysine. CHO and CHO-Le^a cells were bound for 2 hours. After careful and thorough washes, the cells were stained with Hoechst (nuclei) and phalloïdin (actin). Pictures were taken at ×63 magnification with a Zeiss Axiovert 200 microscope.

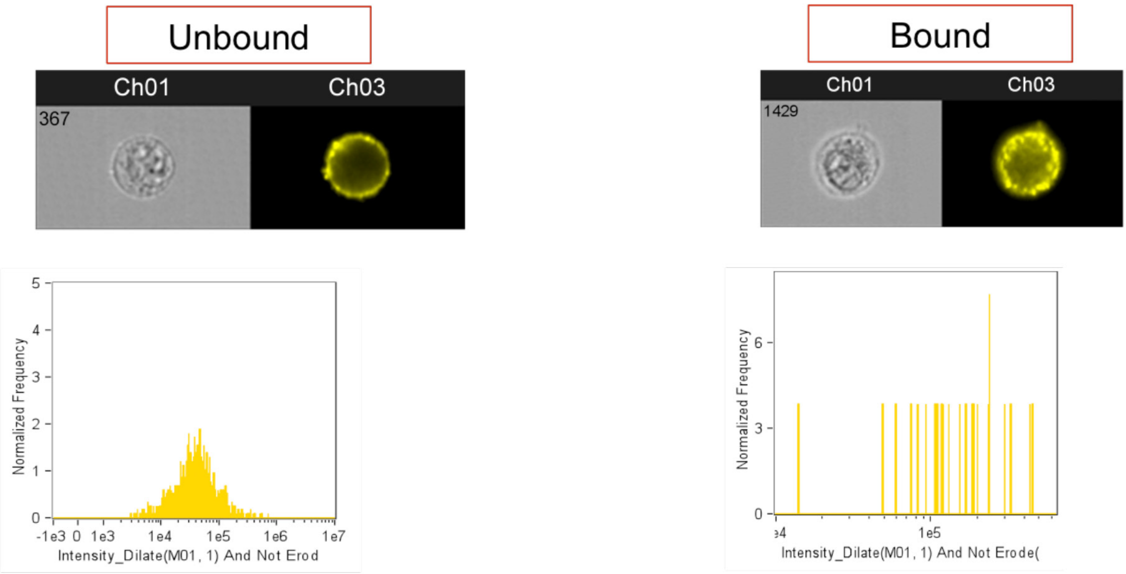

Intensity_Dilate(M01, 1) And Not Erode(M01, 6)_Ch03

Population	Count	%Total	%Gated	Mean	CV
PE+ & cells isolees & focus	1163	21.7	100	51755.66	104

Intensity_Dilate(M01, 1) And Not Erode(M01, 6)_Ch03

Population	Count	%Total	%Gated	Mean	CV
PE+ & cells isolees & focus	26	0.95	100	173957.93	63.56

Supplementary Figure S10: The cells that bind to prolectin under shear flow conditions are those having the highest expression of prolectin ligands. DU-145 were injected into Prolectin-coated BioFlux channels. Unbound cells were washed away with PBS (Ca,Mg) and collected in the output well. Bound cells were detached by trypsin treatment. Both bound and unbound cells were stained with Prolectin/Strep-PE tetramers and analyzed with the Amnis Image Stream X II flow cytometer.

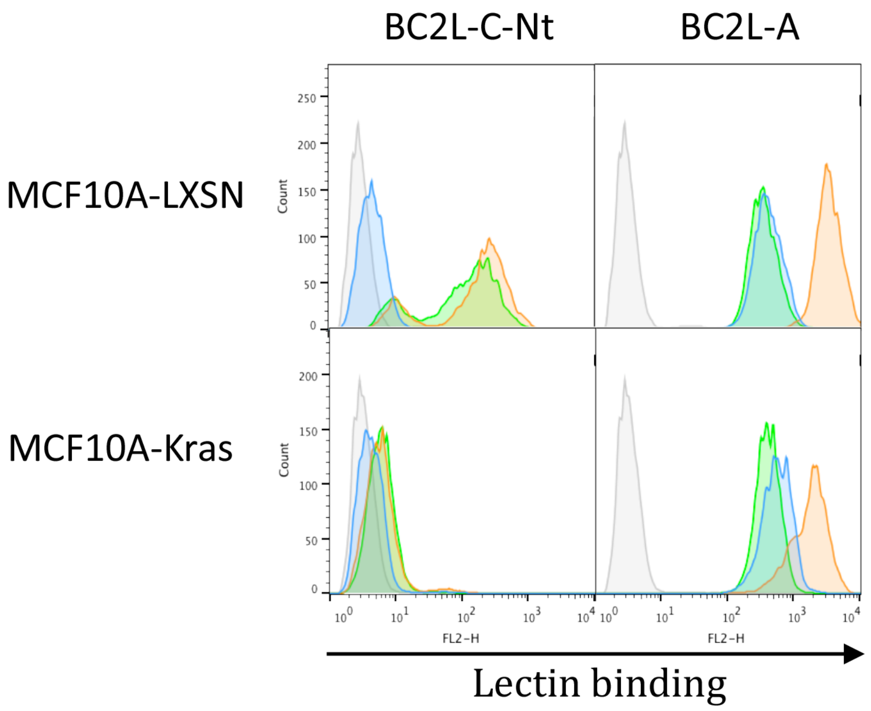

Supplementary Figure S11: Inhibition of fucosylation by 2F-Fuc treatment and increase of mannosylation by KFN treatment on MCF10A cells. MCF10A-LXSN and MCF10A-Kras(v12) were treated with DMSO (green), 5 µM kifunensin (orange) or 400 µM 2 fluoro-fucose (blue) during four days. Cells were then harvested, probed with BC2L-C-Nt-biot (to stain fucosylated structures) or BC2L-A-biot (to stain high mannose structures) followed by streptavidin-PE and analyzed on FACSCalibur flow cytometer.

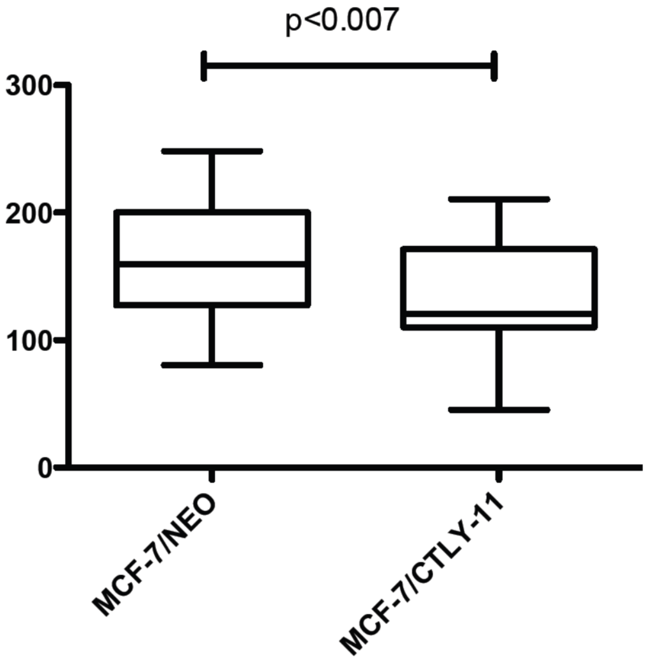

Supplementary Figure S12: Interaction of MCF-7 cells with prolectin-expressing fibroblasts. Rat6-Neo and Rat6-CTLY11 or Rat6-CTLY11 in presence of 25 mM galactose or fucose were injected in BioFlux channels covered with a layer of MCF-7 cells under a pressure of 0,05 Dyn/cm2. Films of 1 minute (600 pictures) were acquired on a Leica DMI 6000B microscope using the Metamorph software. Chronophotographic images, corresponding to 10 pictures segments of the films, were constructed using the FiJi software and distances covered by individual cells were measured. Student *t*-tests were performed to assess statistical significance.

FUT1: Forest plot

Study code	No. patients	No. AE	p-value	HR
Rosetta2002	295	122	0.0293	1.21
GSE1378	59	27	0.4875	1.15
GSE2603	82	27	0.3394	0.83
GSE1456	159	50	0.9810	1.00
GSE2034	286	107	0.1812	1.14
GSE2741	50	13	0.6305	0.87
GSE3143	158	50	0.1144	1.26
E_TABM_158	112	42	0.2798	1.19
GSE4922	249	89	0.9512	0.99
GSE8757	171	56	0.5911	0.93
GSE7390	198	91	0.3567	1.11
GSE6532	393	139	0.0178	1.27
GSE5327	58	11	0.3465	1.32
E_UCON_1	135	65	0.5270	0.93
GSE7378	54	9	0.4091	1.30
GSE7849	75	14	0.3632	1.27
GSE9893	155	57	0.8295	0.97
GSE9195	77	13	0.2355	0.72
GSE11121	200	46	0.0716	1.34
GSE10510	134	96	0.2080	0.88
GSE16391	55	55	0.8571	0.97
GSE12093	136	20	0.1228	1.61
GSE19615	115	14	0.8843	1.04
GSE17907	39	17	0.2695	0.78
GSE22219	216	82	0.0021	1.37
GSE20711	85	36	0.6177	0.92
GSE26971	258	58	0.0636	1.29
GSE25055	309	65	0.5549	0.94
GSE20685	296	73	0.0705	1.27
GSE21653	252	83	0.7601	0.97
GSE16987	147	10	0.2712	0.67
GSE33926	51	12	0.7516	1.09
GSE45255	41	14	0.8763	0.96
**Pool**	**5100**	**1663**	**0.0017**	**1.08**

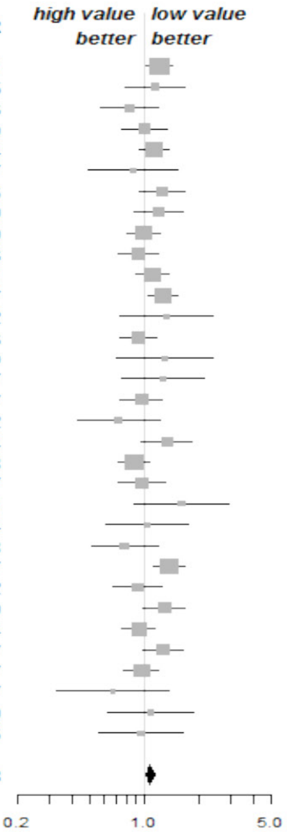

FUT3: Forest plot

Study code	No. patients	No. AE	p-value	HR
Rosetta2002	295	122	0.0133	1.21
GSE1378	59	27	0.0043	1.68
GSE2603	82	27	0.8019	0.94
GSE1456	159	50	0.4981	1.12
GSE2034	286	107	0.8308	0.98
GSE2741	48	12	0.1662	1.37
GSE3143	158	50	0.7653	0.94
E_TABM_158	112	42	0.3577	1.28
GSE4922	249	89	0.8314	1.03
GSE8757	171	56	0.7560	0.96
GSE7390	198	91	0.9424	0.99
GSE6532	393	139	0.2348	1.13
GSE5327	58	11	0.5769	0.82
E_UCON_1	135	65	0.8349	1.03
GSE7378	54	9	0.4673	0.67
GSE7849	75	14	0.5491	1.18
GSE9893	155	57	0.5503	0.92
GSE9195	77	13	0.7576	1.12
GSE11121	200	46	0.3274	0.85
GSE10510	134	96	0.6783	1.05
GSE16391	55	55	0.3649	1.18
GSE12093	136	20	0.5373	0.84
GSE19615	115	14	0.0194	1.94
GSE17907	39	17	0.3470	0.79
GSE22219	216	82	0.0010	1.37
GSE20711	85	36	0.0983	1.38
GSE26971	258	58	0.7507	1.06
GSE25055	309	65	0.0042	1.57
GSE20685	296	73	0.4522	1.11
GSE21653	252	83	0.2204	1.16
GSE16987	147	10	0.0529	1.71
GSE33926	51	12	0.0223	2.38
GSE45255	41	14	0.4511	1.26
**Pool**	**5098**	**1662**	**< 0.0001**	**1.12**

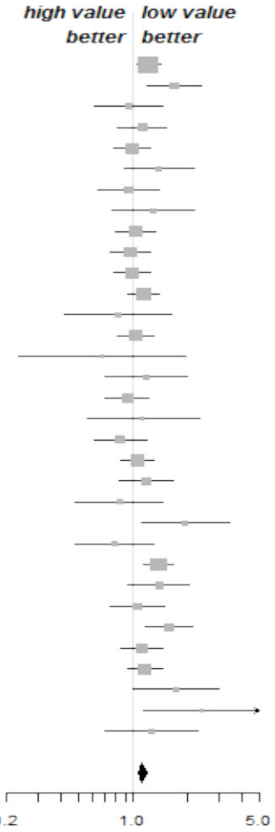

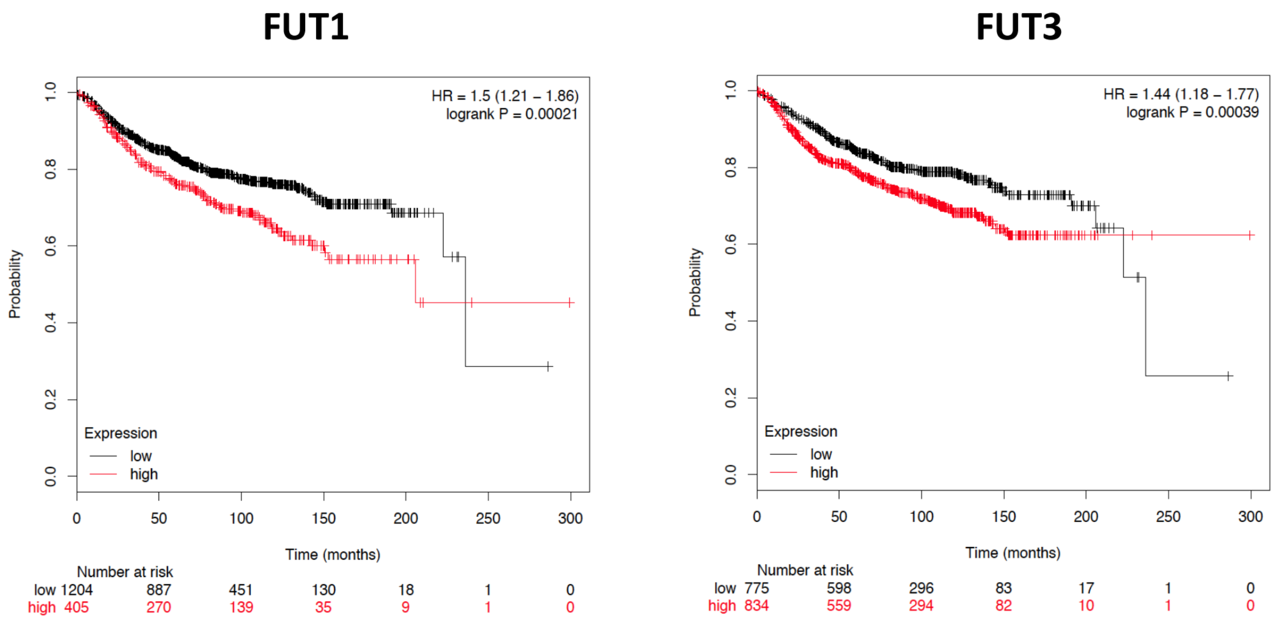

Supplementary Figure S13: Prognostic value of *FUT1* and *FUT3* in breast cancer. (**A**) Computational analysis of breast cancer patients data were performed using the online platform bc genexminer (http://bcgenex.centregauducheau.fr). *FUT1* and *FUT3* expression are grouped into high and low level expression by comparison with the median expression of each gene. Analysis shown was done on all the patients irrespective of their nodal or estrogen receptor status. (**B**) Computational analysis was also performed using the online platform KM plotter (http:/ /kmplot.com/ analysis/index.php?p=service&cancer=breast) using the Jetset best probe set and automatic selection of the best cutoff to split patients. Analyses are shown on all patients and the time represents distant metastasis free survival.

Supplementary Table S1: Lectins

Lectin	Species	Specficity	Main Ligands	Working concentration
PA-IL	*Pseudomonas aeruginosa*	Gal	Gb3	20 µg/ml
RSL	*Ralstonia solanacearum*	Fuc	Blood group antigens	0,1 µg/m
BC2L-A	*Burkholderia cenocepacia*	Man	Dimannoside	2 µg/ml
BC2L-C-Nt	*Burkholderia cenocepacia*	Fuc	H type 1/3, Lewis Y	5–10 µg/ml
UEA-I	*Ulex europeaus*	Fuc (α 1, 2)	H type 2	1 µg/ml

Supplementary Table S2: Primers and probes used for qRT-PCR analysis

Gene	Acces. nb	Forward primer	Reverse primer	Probe
FUT1	NM_000148	AGGTATAAACACAC CCTCTGTGCTT	GAGTTCAGGGACAG ACAGT GGTT	AAACTGGCAGGTACCGT GCTCATTGC
FUT3	NM_000149	GGGATCCCTTTTCG TCACACT	CGAACTGGTCTAAG CCTTGCA	AGGTGACCTACAGGCTC CGCTCGA
CDH1	NM_004360.3	GGGCGAGTGCCAA CTGGACC	CCAGCGGCCCCTTC ACAGTC	/
SNAI1	AF155233.1	GACCCCAGTGCCTC GACCACTA	CAGCAGGTGGGCCT GGTCGTA	/
SNAI2	NM_003068.4	GACCCGCTGGCAA GATGCCG	GCTGGGCGTGGAAT GGAGCA	/
b-Actin	X00351.1	AGAAAATCTGGCAC CACACC	CAGAGGCGTACAGG GATAGC	/

Supplementary Table S3: Synthetic oligosaccharides used for the ELISA

**Poly-Acrylamide (PAA)**	**A type 2**	GalNAcα3[Fucα2]Galβ4GlcNAcβ-R1
	**A tri**	GalNAcα3[Fucα2]Galβ-R1
	**A di**	GalNAcα3Galβ-R1
	**B type 2**	Galα3[Fucα2]Galβ4GlcNAcβ-R1
	**B tri**	Galα3[Fucα2]Galβ-R1
	**B Di**	Galα3Galβ-R1
	**H Di**	[Fucα2]Galβ-R1
	**H type 2**	[Fucα2]Galβ4GlcNAcβ-R1
	**H type 3**	[Fucα2]Galβ3GalNAcα-R1
	**Le^{a}**	Galβ3[Fucα4]GlcNAcβ-R1
	**Le^{b}**	[Fucα2]Galβ3[Fucα4]GlcNAcβ-R1
	**Le^{x}**	Galβ4[Fucα3]GlcNAcβ-R1
	**Le^{y}**	[Fucα2]Galβ4[Fucα3]GlcNAcβ-R1
**Human serum albumine (HSA)**	**A tri**	GalNAcα3[Fucα2]Galβ-R2
	**A hepta**	GalNAcα3[Fucα2]Galβ3[Fucα4]GlcNAcβ3Galβ4Glcβ-R2
	**B tri**	Galα3[Fucα2]Galβ-R2
	**H type 1**	[Fucα2]Galβ3GlcNAcβ-R2
	**H type 2**	[Fucα2]Galβ4GlcNAcβ-R2
	**Le^{a}**	Galβ3[Fucα4]GlcNAcβ-R2
	**Le^{b}**	[Fucα2]Galβ3[Fucα4]GlcNAcβ-R2
	**Le^{x}**	Galβ4[Fucα3]GlcNAcβ-R2
	**Le^{y}**	[Fucα2]Galβ4[Fucα3]GlcNAcβ-R2

Supplementary Table S4: Surface expression of Le^{x} and Le^{y} on the tumor cell lines used for the prolectin binding experiments

**Cell lines**	**Le^{x} (3E1)**	**Le^{y} (12–4Le)**
MCF-7	+ + +	+++
HT-29	+ + +	+
OVCAR-3	+++	+++
DU-145	++	+/–
